# Brucellae as resilient intracellular pathogens: epidemiology, host–pathogen interaction, recent genomics and proteomics approaches, and future perspectives

**DOI:** 10.3389/fvets.2023.1255239

**Published:** 2023-10-09

**Authors:** Ali Sobhy Dawood, Alyaa Elrashedy, Mohamed Nayel, Akram Salama, Aizhen Guo, Gang Zhao, Samah Attia Algharib, Ahmed Zaghawa, Muhammed Zubair, Ahmed Elsify, Walid Mousa, Wanhe Luo

**Affiliations:** ^1^Engineering Laboratory for Tarim Animal Diseases Diagnosis and Control, College of Animal Science and Technology, Tarim University, Alar, Xinjiang, China; ^2^The State Key Laboratory of Agricultural Microbiology, College of Veterinary Medicine, Huazhong Agricultural University, Wuhan, China; ^3^Department of Medicine and Infectious Diseases, Faculty of Veterinary Medicine, University of Sadat City, Sadat City, Egypt; ^4^Key Laboratory of Ministry of Education for Conservation and Utilization of Special Biological Resources in the Western China, School of Life Sciences, Ningxia University, Yinchuan, China; ^5^National Reference Laboratory of Veterinary Drug Residues (HZAU) and MAO Key Laboratory for Detection of Veterinary Drug Residues (HZAU), Wuhan, China; ^6^Department of Clinical Pathology, Faculty of Veterinary Medicine, Benha University, Toukh, Egypt; ^7^Key Laboratory of Veterinary Biological Engineering and Technology, Institute of Veterinary Medicine, Jiangsu Academy of Agricultural Sciences, Nanjing, China

**Keywords:** emerging *Brucella* pathogens, contagious disease, prevention and control, *Brucella* vaccines, genomics and proteomics approaches, future perspectives

## Abstract

Brucellosis is considered one of the most hazardous zoonotic diseases all over the world. It causes formidable economic losses in developed and developing countries. Despite the significant attempts to get rid of *Brucella* pathogens in many parts of the world, the disease continues to spread widely. Recently, many attempts proved to be effective for the prevention and control of highly contagious bovine brucellosis, which could be followed by others to achieve a prosperous future without rampant *Brucella* pathogens. In this study, the updated view for worldwide *Brucella* distribution, possible predisposing factors for emerging *Brucella* pathogens, immune response and different types of *Brucella* vaccines, genomics and proteomics approaches incorporated recently in the field of brucellosis, and future perspectives for prevention and control of bovine brucellosis have been discussed comprehensively. So, the current study will be used as a guide for researchers in planning their future work, which will pave the way for a new world without these highly contagious pathogens that have been infecting and threatening the health of humans and terrestrial animals.

## 1. Introduction

Brucellosis is a zoonotic infection caused by intracellular *Brucella* pathogens (1). Twelve species are now considered in the genus *Brucella* that infect different domestic and wildlife animal species (2). Among them, six *Brucella* species have been classified according to their pathogenicity and natural hosts as *Brucella* (*B.*) *abortus* (cattle), *B. melitensis* (sheep and goats), *B. suis* (pigs), *B. canis* (dogs), *B. ovis* (rams), and *B. neotomae* (Common voles and desert wood rat) (3, 4). *B. melitensis* and *B. abortus* are the most significant species in animals and humans (5). Later, two new *Brucella* spp., namely, *B. pinnipedialis* (walruses and seals) and *B. ceti* (dolphins, porpoises, and whales), have been reported to infect marine mammals (6). Brucellosis is a major health concern for animals because it causes significant economic losses in developing countries across the world in the form of abortion, stillbirth, delayed conception, retained placenta, reduced milk production, contagious epididymitis, and the cost of treatment and productivity loss (7) (Table 1). Abortion and infertility are the most common clinical symptoms of animal brucellosis, and they are not specific in nature. Abortion always occurs during the first pregnancy, but it is less commonly present after that due to persistent immunity (24). In addition, Xie et al. proved that there are three parameters, including animal species, vaccination dose, and immunization route, that were statistically linked to the occurrence of abortion as an adverse reaction to *Brucella* vaccination (25). Since it is easily transmissible by exposure to aborted fetuses, placentae, vaginal fluids, and milk, congenitally and/or venereally, the disease is very contagious in all circumstances (26).

**Table 1:** Estimated worldwide economic losses of brucellosis.

*Brucella* species	Economic losses	Economic losses	Date of study	Animal species	References
*B. abortus*	USD 32 million annually	Brazil	2002	Cattle	(8)
	Abortion 15%. Perinatal mortality rates 10%. Mortality risk for infected cows 1%. Temporary infertility 20%. Milk losses 15%. Meat losses 5% Replacement costs of infected cows, heifers, and bulls. Veterinary costs.		2013		(9)
Bovine brucellosis	USD 60 million annually	Argentina	2002	Cattle	(10)
Bovine brucellosis	Reduced milk production Abortion, increase the inter-calving period Repeat breeding Cost of veterinary examination and treatment	Khartoum State, Sudan	April to October 2012	Cattle	(11)
*B. abortus* and *B. melitensis*	Reduced milk production	Dushanbe, Tajikistan.	May to October 2011	Cattle	(12)
*B. abortus, B. melitensis,* and *B. ovis*	USD 62,926,060.84 annually	Malaysia	2012	Cattle, buffaloes, goats, and sheep	(13)
Caprine brucellosis	USD 50,391.13 annually		July to September 2011.	Goats	(14)
*B. abortus*	The imbalance between the sale of sick animals and the purchase of healthy animals of a similar level of production. The losses were caused by a reduction in the volume of production during the quarantine.	Ecuador	2015 to 2017	Dairy cattle	(15)
*B. abortus* and *B. melitensis*	The cost spent on compensation (USD 21 million) for brucellosis animals. Testing animals $24 million annually	Kazakhstan	2015	Cattle and small ruminants	(16)
*Brucella* spp.	The economic cost of brucellosis in yaks and control program (US$ 521,043).	China	April to May 2015	Yaks	(17)
Bovine brucellosis	USD 6.8 per cattle USD 18.2 per buffalo USD 0.7 per sheep USD 0.5 per goat USD 0.6 per pig.	India	2015	Cattle, sheep, goats and pig	(18)
	Financial costs USD 3.43 billion		2018	Cattle	(19)
	Reduced milk yield (46%) Extended calving interval (18%) Treatment cost of abortion (14%) Treatment cost of metritis/endometritis (8%)		2003 to 2005	Cattle and buffalo	(20)
*Brucella* spp.	Reproductive loss: Losses due to abortion Losses due to increased inter-calving/kidding/lambing/farrowing period Production losses: Direct loss in milk production Direct loss in carcass weight Losses due to mortality in adult animals that were aborted. Draught power loss		1998 to 2014	Cattle, buffalo, Goats, Sheep and Pigs	(21)
*Brucella* spp.	Drop in milk production, infertility, sale value, abortion, weak calf and lamb, swollen joints, and cost of treatment.	Baringo County, Kenya	2020	Sheep, Goats, Cattle, and Camels	(22)
*Brucella* spp.	Cost benefits analysis of mass vaccination strategy (USD 1.75 per adult female animal).	Northern Iraq	20-year period from the implementation of the vaccination program.	Sheep and goats	(23)

Human infections mostly occur after contact with infected cattle, sheep, goats, and camels. In humans, brucellosis is characterized by a variety of non-specific symptoms such as malaise, lethargy, arthritis, and fever. These symptoms are mostly chronic lasting for years, and treatment needs to ensure compliance with a costly and prolonged therapy (27). It also can be manifested as a neurological disorder (28). People can get the infection by contact with infected animals during their usual work (occupational) as in the case of veterinarians, abattoir workers, and livestock keepers. Consumption of unpasteurized milk is also regarded as a common source of infection (29). Although pasteurizing milk kills *Brucella* and prevents infection in humans, it is not commonly performed in some communities with a widespread lack of public awareness about the danger of drinking raw milk (30). In addition, human-to-human transmission was reported via breastfeeding, hardly through sexual intercourse, blood transfusions, organ transplantation, and transplacentally (31). *Brucella* infections are more likely to be prevented and controlled when immunization is combined with adequate preventive measures (32).

As a result of increasing the severity of brucellosis, in this study, we described its global distribution and possible predisposing factors that facilitate the infection’s spreading. The immune response and how *Brucella* can evade the host immune system, as well as the recent trends and advances in the diagnosis, vaccination, treatment, prevention, and control of bovine brucellosis, have been discussed comprehensively. Subsequently, this review can be used as a guide for veterinarians and health workers to stop the further spread of this contagion all over the world.

## 2. Brucellosis worldwide

Brucellosis is one of the most common zoonotic diseases that increase poverty levels in poor nations (1). It has a negative influence on the world economy and public health. Annually, from 5 to 12.5 million human cases of brucellosis are reported, making it one of the most serious zoonoses on the globe (33). Human *Brucella* seroprevalence has been reported in many countries all over the world, especially among a variety of highly occupational groups as follows: China (15.5%) (34); India (1.83%) (35); Pakistan (17%) (36); Malaysia (5.4%) (37); Saudi Arabia (33.9%) (38); Greece (3%) (39); Egypt (31.3%) (40); South Sudan (33.3%) (41); Nigeria (17.6%) (42); Cameroon (5.6%) (43); Kenya (32.3%) (44); Uganda (17%) (45), and Tanzania (1.41%) (46) (Supplementary Table S1).

The annual incidence rate of human brucellosis is between 0.5/100,000 and 70/100,000, depending on the area of study (47), while the prevalence of brucellosis in animals ranges from 0.2 to 20%, depending on the location and species (48) (Supplementary Table S1). Azerbaijan is now ranked 13th in the world for the incidence of human brucellosis, with an estimated yearly incidence of over 50 cases per million (49). Human brucellosis has recently been linked to raw milk consumption and the RB51 vaccination status (50, 51). Therefore, the raw milk movement in the United States has been controlled, and numerous states have approved legislation that restricts the selling of raw milk and raw milk products to customers. Nowadays, less than 100 cases are recorded annually in the United States, with the majority coming in the South and Southwest from illegally imported soft cheeses (unpasteurized) from Mexico (52). In Tanzania, the upfront cost of pasteurization facilities is now unaffordable; hence, alternative management techniques are regarded as more cost-effective. In a Tanzanian investigation, 56% of the inspected milk samples (*n* = 59) tested positive for brucellosis (53).

*Brucella* is regarded as a spillover organism that begins to affect other species suddenly. From this point, cattle can be infected with both *B. melitensis* and *B. abortus* (49). In Azerbaijan, milk and blood samples were collected during early lactation in farms with a history of abortions and previous seropositive results for brucellosis. Out of 57 milk samples collected from seropositive cows, 22 samples revealed microbial growth on Farrell’s media with 5% CO2 incubation. On the other hand, 8 milk samples revealed growth in the absence of CO2. According to biotyping classification, these 22 and 8 samples were classified as *B. abortus* and *B. melitensis*, respectively. By using multiple-locus variable number tandem repeat analysis (MLVA) and matching the two types of *Brucella*, *B. abortus* strains were matched with *B. abortus* strains isolated in East Europe, Central Asia, and China. *B. melitensis* strains had a new genotype circulating among cattle, sheep, and goats in Azerbaijan, and these strains belong to the American clade that is hardly identified in the region (49). In Mongolia, seropositive camel herds were closely associated with cattle infection (54). In China, the total prevalence of bovine brucellosis was 1.9% according to a meta-analysis conducted over a 10-year period (2008–2018). Northern China has a higher prevalence than Southern China. More specifically, Jilin province has the highest prevalence of more than 30% (55). During previous Pakistani research, RBPT analysis of cow and buffalo samples revealed that 170 serum samples (6.3%) and 47 herds (18.6%) were seropositive for brucellosis (56). In a recent meta-analysis of animal diseases in North Eastern India, bovine brucellosis was shown to be prevalent in 17% of cattle (57). In addition, in India, Pathak et al. examined 481 samples from 296 animals, including milk, blood, vaginal swabs, vaginal discharges, placental tissue, and fetal tissues. Of these samples, 30.4 and 41.6% were positive for brucellosis using RBPT and iELISA, respectively, whereas 27.0% were seropositive by both tests (58). In domestic animals, only Canada, the United States, Japan, Australia, New Zealand, and Western and Central European countries are almost free of both *B. abortus* and *B. melitensis* (59).

On the other hand, the disease has a devastating impact on Africa, the Middle East, Latin America, Eastern Europe, and large parts of Asia (60). In Jordan, Musallam et al. estimated seroprevalence levels of 18.1% in cow herds (61). In Egypt, the prevalence frequency varies greatly across the country (62). *B. melitensis*, *B. abortus*, and *B. suis* are the three zoonotic *Brucella* species found in Egypt. *B. melitensis* biovar 3 is the most frequent *Brucella* isolate in Egypt right now in both livestock and people (63). Hosein et al. studied the outbreak in buffalo caused by *B. melitensis* biovar 3. The study revealed that being free of brucellosis required 6 months, which is considered a long time for transmitting infection to other areas, particularly in unhygienic conditions and husbandry systems that allow mixed populations of different ages and sexes and abortion with a lack of controlled animal movement (64). A novel *Brucella* strain closely related to *B. melitensis* biovar 3 was identified in Croatian cattle during testing within a brucellosis eradication program (65). Chaka et al. discovered that the herd-level prevalence of brucellosis in Ethiopian cattle was 32%, while an overall cattle-level prevalence of 9.7% was recorded dependent on serological tests (66). Moreover, Getachew et al. tested 278 serum samples for brucellosis in dairy animals in Ethiopia by RBPT, indirect ELISA (iELISA), and complement fixation test (CFT). The sensitivity was reported as 89.6, 96.8, and 94%, respectively, and the specificity was reported as 84.5, 96.3, and 88.5%, respectively. Comparing the three tests, iELISA was shown to have the highest sensitivity and specificity (67).

**Figure 1:**
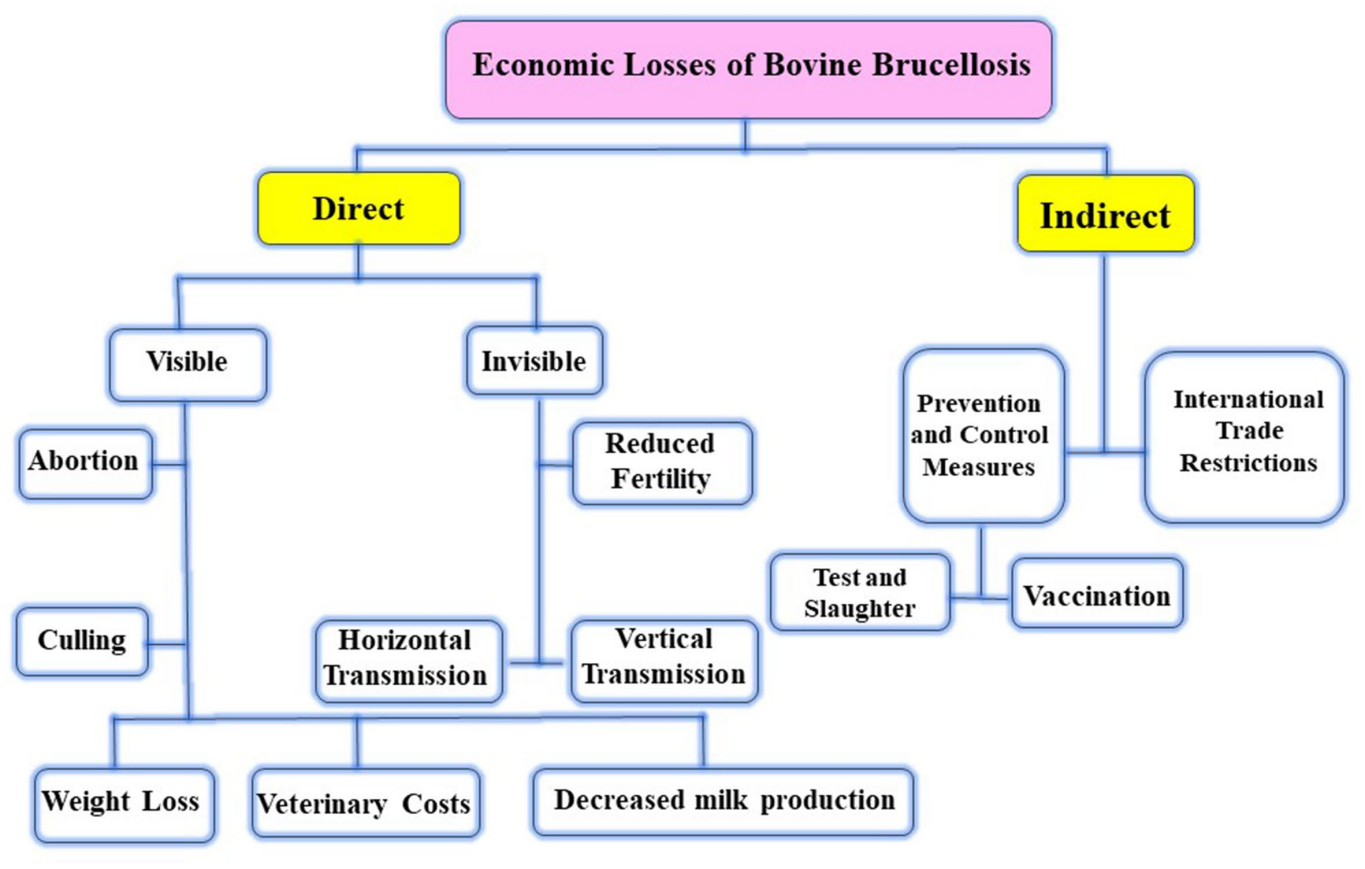
The effect of brucellosis on animals.

In Abuja, Nigeria, Aworh et al. tested 376 cattle for *Brucella* infection. Twenty-one animals were RBPT positive, and two animals gave positive competitive ELISA (cELISA) results (68). Using the milk ring test and iELISA, Kamwine et al. reported a prevalence of 26.5% of *Brucella* in 185 raw milk samples in Uganda (69). In Tanzania, milk samples were used for iELISA-based *Brucella* surveillance, and the study revealed a herd frequency of 44.4% (70). In Ghana, blood samples were collected from 315 cattle and 178 cattle farmers to measure *Brucella* seroprevalence, and the RBPT results demonstrated 22.9 and 10.1% in bovines and humans, respectively (71). Madut et al. demonstrated a high prevalence level of brucellosis in cattle and their owners in Bahr El-Ghazal, Sudan (41). In Ecuador, brucellosis seroprevalence and risk variables have been investigated in dairy and dairy-beef mixed cattle herds, and the results indicated that the true *Brucella* seropositivity was 17.0% (72) (Figure 1).

## 3. Risk and predisposing factors for bovine brucellosis

**Figure 2:**
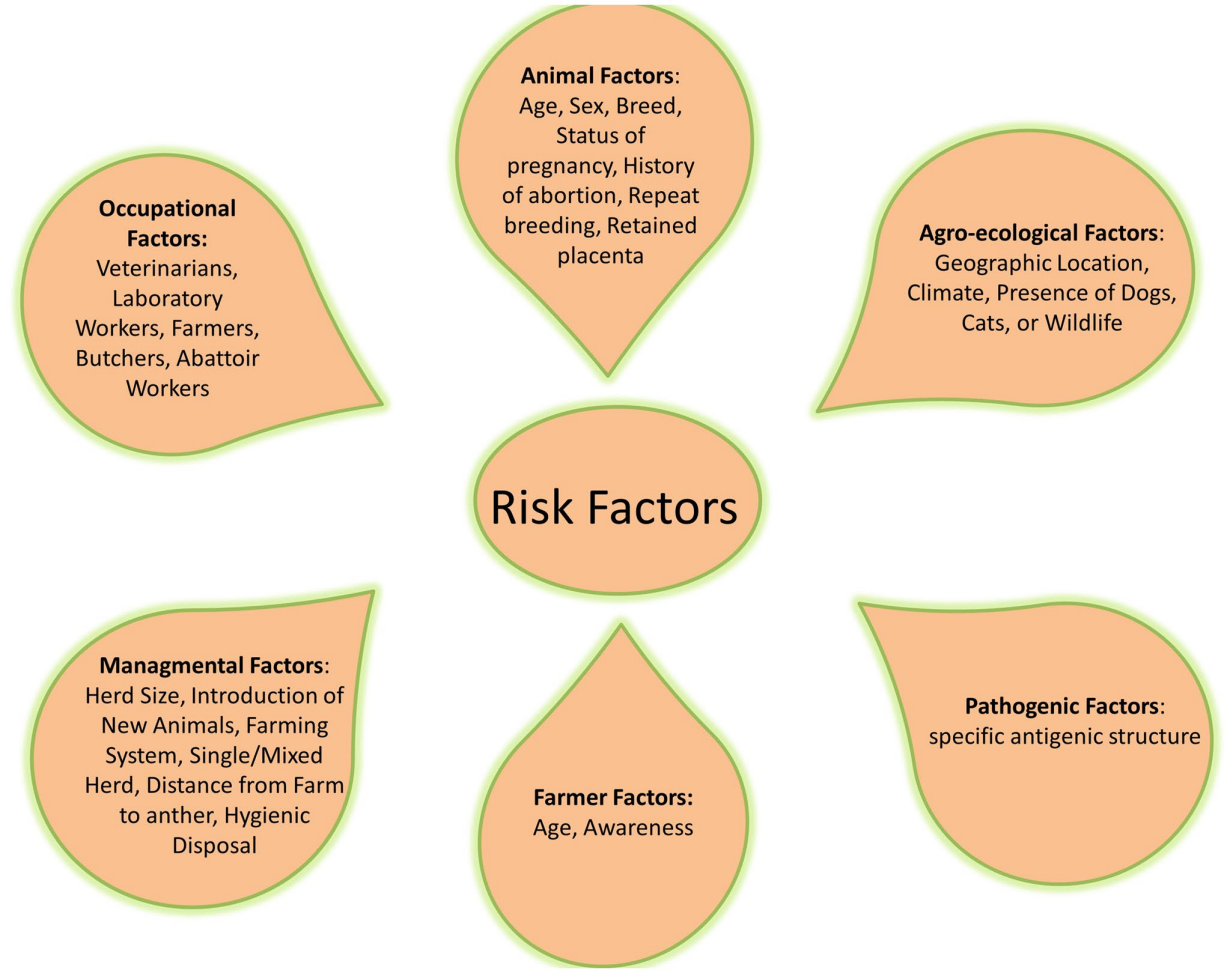
The risk and predisposing factors of brucellosis.

Common risk factors related to the difficulty of brucellosis control are social and political instabilities and inappropriate diagnosis, reporting, and application of control measures. Livestock husbandry systems and their interactions with other animals and wildlife, as well as using unpasteurized dairy products as one of the traditional cultural habits, played a major role in transmitting brucellosis (32, 73–75) (Figure 2).

The outbreaks of brucellosis in animals are increasingly important nowadays because of its asymptomatic features in humans. The follow-up outcomes of asymptomatic brucellosis without treatment were investigated in 3,610 studies from 1990 to 2021. Of which, 13 studies included that during a 0.5- to 18-month follow-up period, 40.3% of the cases remained asymptomatic. Moreover, investigations on brucellosis transmission vary between different geographies and populations (76). Moreover, many studies have detected substantial correlations among species, gender, age, and breed of animals with seropositivity (77–79). Lack of clean water, insufficient manure collection, neglected cleaning, bad management of aborted materials, the introduction of new animals from infected herds or herds with unclear status, and mixed herds are among the other risk factors that have been highlighted (80) (Table 2). Also, various studies have shown that brucellosis has a significant influence on Middle Eastern countries, where contaminated milk products have been responsible for several outbreaks of human brucellosis during the previous two decades (75, 91). The knowledge and awareness of farmers about brucellosis significantly reduce *Brucella* infection in animals (98). The large-scale organized dairy farms have higher incidence rates than the individual native animals, which could be related to the disease’s higher prevalence in exotic and cross-bred animals compared to native cattle (77). Disease transmission usually occurs through natural mating/artificial insemination and physical contact and exposure to affected animals (99). The microorganism is more vulnerable to infection in sexually mature pregnant cattle than in sexually immature cattle of either sex, and susceptibility increases as the pregnancy progresses (100). Most animals get infected and harbor infection for the rest of their lives.

**Table 2:** The risk factors of brucellosis.

Risk factors		Significance association with brucellosis	References
Animal Factors	Species	Cattle are more likely to be infected than buffalos	(81)
	Age of animals	Sero-positive animals are more likely to be older age than calves	(82)
	Sex	Female dairy animals are more likely to be infected than males	(83)
	Breed	Purebred animals are more likely to be seropositive than native breeds	(84)
	History of abortion	The history of abortion is related to seropositive cases	(85)
	Status of pregnancy	The late stage of pregnancy is more likely to be seropositive	(86)
	History of retained placenta	The history of retained placenta is linked with seropositive cases	(87)
Pathogen factors		Intracellular replication Inhibition of bactericidal activity	(88)
		Intracellular replication Phagocytosis inhibition	(89, 90)
Occupational factors		Veterinarians, laboratory workers, farmers, butchers, abattoir workers	(91)
Management factors	Herd size	Larger herds are positively associated with seropositivity	(92)
	Mixed herd	Cattle being housed with goats and/or sheep are more likely to be seropositive	(92)
	Breeding method	Breeding by artificial insemination is positively co-related with seropositivity	(92, 93)
	Distance between herds	Herds located close to one another are positively associated with seropositivity	(92)
	Introduction of a new animal from an unknown source	The introduction of new animals from unknown sources is positively associated with seropositivity	(77)
	Clean drinking water	Lack of clean drinking water for animals is positively associated with seropositivity	(94)
	Clean and hygiene	Insufficient manure removal and dirtiness in farms are positively associated with seropositivity	(94)
	Routine milk diagnosis	Herds that are not routinely tested for *Brucella* infection were positively co-related with seropositivity	(30)
Human Factors	Age of owner	Cattle and buffalo belonging to older age group owners (above 40 years) are positively associated with seropositivity	(95)
	Awareness	Cattle and buffalo belong to farmers who have knowledge and awareness about diseases, particularly brucellosis is negatively correlated with seropositivity.	(58)
Agro-ecological factors	Origin	Sero-positivity differs in different parts of a country	(96)
	Presence of dogs, cats, and wildlife	Their presence is suspected as a source of infection	(97)

The prevalence of disease and the difficulty of controlling infection in a population are closely related to herd size and animal density (101). Animal species also affect disease susceptibility, and so a survey carried out for cattle, camels, sheep, and goats in a pastoral area in Isiolo County, Kenya, using RBPT and iELISA showed that camels and cattle have a greater incidence of brucellosis than sheep and goats (102).

According to the chairman of the Egyptian Chamber of Food Industries, social misconceptions about the nutritional quality of pasteurized milk have led to a decrease in the consumption of commercial milk products. More and more people buy fresh, unprocessed raw milk from small-scale dairy producers to account for up to 80% of the total milk industry (up to 4 billion liters per year) (103). In Fayoum province, Upper Egypt, Abdel-Hamid and her group have investigated the risk factors and molecular genotyping of *B. melitensis* strains. From a total of 183 cattle cases, 78 (42.6%) were seropositive cattle cases. All seropositive cattle were female, and 85.9% (*n* = 67) of them were older than 2 years. One-third of the seropositive cattle (*n* = 28) were reared alone. Nine of the seropositive cattle (11.5%) and one of the seronegative cattle (0.9%) had abortions. According to the socio-demographic variables, uneducated individuals, workers of an animal-related occupation, or owners of household cattle had a greater risk of brucellosis. In addition, in a multivariable model, soft cheese consumption was 2.3 times more likely to be responsible for brucellosis cases than controls (*p* 0.03) (104).

In France, the country has been declared free since 2005. So, a study was performed to measure the impact of veterinarians and all local institutional stakeholders on the compulsory notification of bovine abortions by farmers. The results showed that the proportion of notifying farmers was influenced by the number of veterinarians per practice and the veterinary practice’s membership of a technical association (105). In South China, the major source of brucellosis for human infection was goats, so a cross-sectional study was performed to investigate the true prevalence and risk factors for goat brucellosis in Ningxiang County, South China. The herd prevalence in commercial goat farms was 4.5% with nine potential risk factors identified by logistic regression analysis (*p* < 0.2). Among these factors, introduction in the past 12 months, self-breeding, and safe disposal of sick or dead animals were the factors with the strongest association with disease presence (106). In Nepal, another cross-sectional study was performed to investigate brucellosis in sheep and goats. At the farm level, 31.6% of sheep farms and 3.3% of goat farms were seropositive to brucellosis. Age > 1.5 years (older sheep; *p* 0.02) and herd size >100 (larger herds; *p* 0.03) were demonstrated as significant risk factors for brucellosis in the sheep population. While in the goat population, none of them was identified as a significant factor. Because sheep were frequently moved for grazing and selling, a management scheme for the sheep population should be implemented with strict biosecurity (107).

## 4. Immunity

### 4.1. Innate immunity

The first line of defense against pathogen attacks is the innate immune system. Anatomical barriers (skin and internal epithelial layers), secretory molecules (chemokines and cytokines, complement system, and opsonins), cellular populations such as phagocytes [neutrophils, monocytes/macrophages, and dendritic cells (DCs)], and innate lymphocyte subsets [natural killer (NK) cells and T cells] are all parts of the innate immune system (108). *Brucella* antigenic components, such as lipopolysaccharide (LPS), Type IV secretion system (T4SS), BvrR/BvrS, outer membrane protein (Omp), proline racemase subunit A (PrpA), and Btp1, regulate the unique techniques that enable *Brucella* to elude the immune system. Intracellular survival, slowed phagocytosis, antibacterial action, and tumor necrosis factor-alpha (TNF-α) production are among these mechanisms (109).

**Figure 3:**
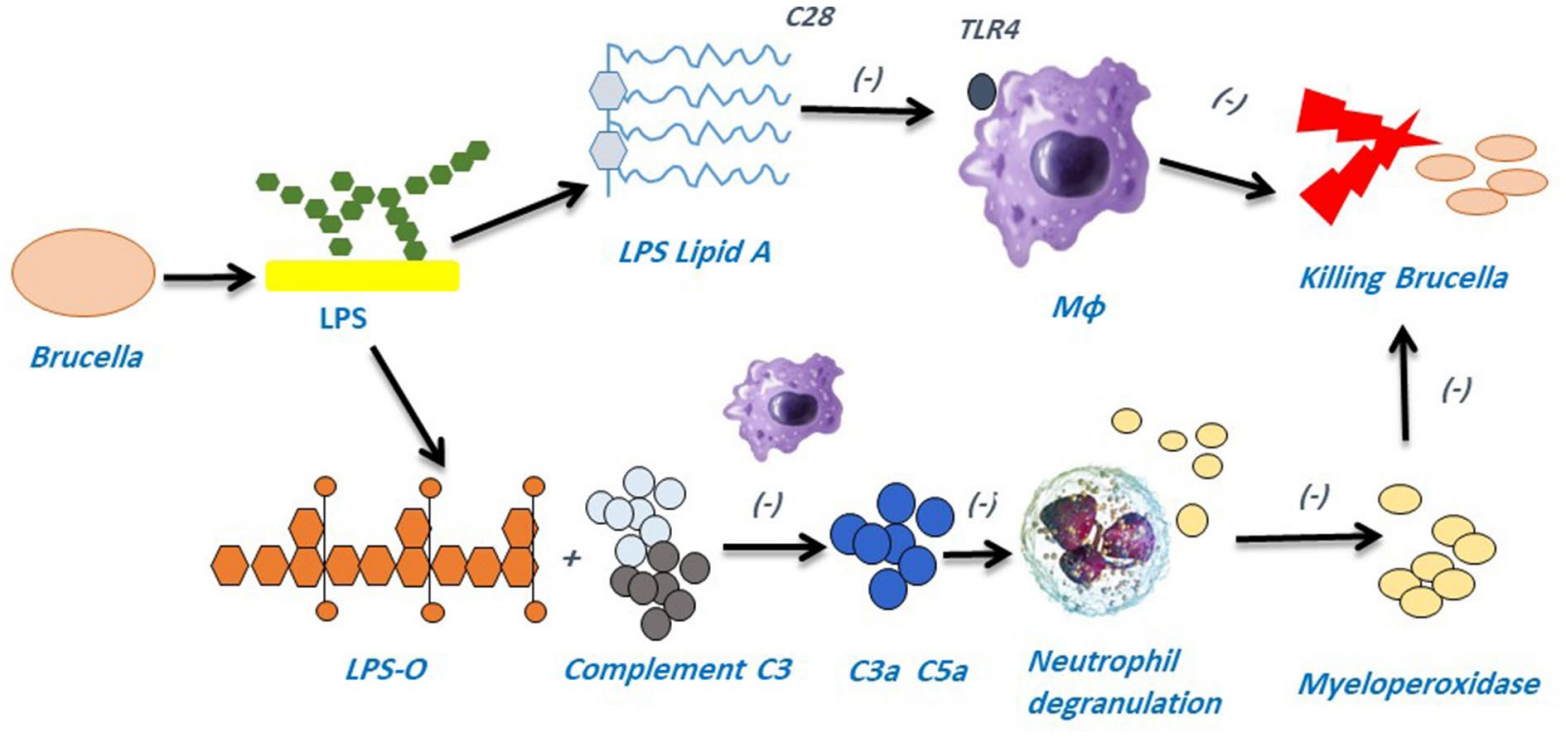
LPS is a pivotal component of *Brucella’s* pathogenicity and aids in the pathogen’s ability to evade the host immune system. TLR4 is not recognized by *Brucella* LPS’s acetyl side chain (C28), which prevents the host immune system from being monitored. The O-chain found in *Brucella* LPS can block complement C3 from producing C3a and C5a, which in turn can stop neutrophils from degranulation and releasing myeloperoxidase (MPO) and other lysosomal chemicals that could otherwise be collected by the host immune system.

*Brucella* are considered resilient pathogens that elude innate immunity and stimulate polymorphonuclear cells (PMNCs) to enhance their microbicidal activity, allowing leukocytes to live longer and resist phagocyte mechanisms (110). It was discovered that eliminating PMNCs before inducing adaptive immunity helped mice to get rid of bacteria, which in turn proved that neutrophils suppress the host immune response against *Brucella* (111). The interaction of pattern recognition receptors (PRRs) and pathogen-associated molecular patterns (PAMPs) allows host cells to recognize threatening compounds and encourages the body to figure out how to fight pathogens (112). *Brucella* LPS influences the complement system; thereby, the O-chain lacking a free hydroxyl is beneficial for connecting with C3, which suppresses the formation of C3a and C5a by associating with *Brucella* LPS’s unique O-chain and avoiding host immunity (113) (Figure 3). *Brucella* flagellin is also crucial for immune evasion and lacks the distinctive characteristic identified by toll-like receptor-5 (TLR5) (114). In addition, host peroxiredoxin 6 (Prdx6), a bifunctional protein with peroxidase and phospholipase activities, has a function in *Brucella* infection by increasing the intracellular survival of the *B. suis* S2 strain (115).

### 4.2. Intracellular life of *Brucella* pathogens

Intracellular pathogens are characterized by many occult pathways that need further experimental work to overcome the growing threat of many zoonotic pathogens (116). In parallel, the growing concern regarding their resistance to different antimicrobials made further studying of their interaction with host cells increasingly important (117–119). *Brucella* has a multistage, complex intracellular replication process that affects the endocytic, secretory, and autophagic compartments through type IV secretion system (T4SS)-mediated delivery of bacterial effectors. These effectors enhance the conversion of the initial endosome-like *Brucella*-containing vacuole (eBCV) into a replication-permissive organelle derived from the host endoplasmic reticulum replicating BCV (rBCV), after that, to an autophagy-related vacuole (aBCV) that mediates bacterial egress (120).

**Figure 4:**
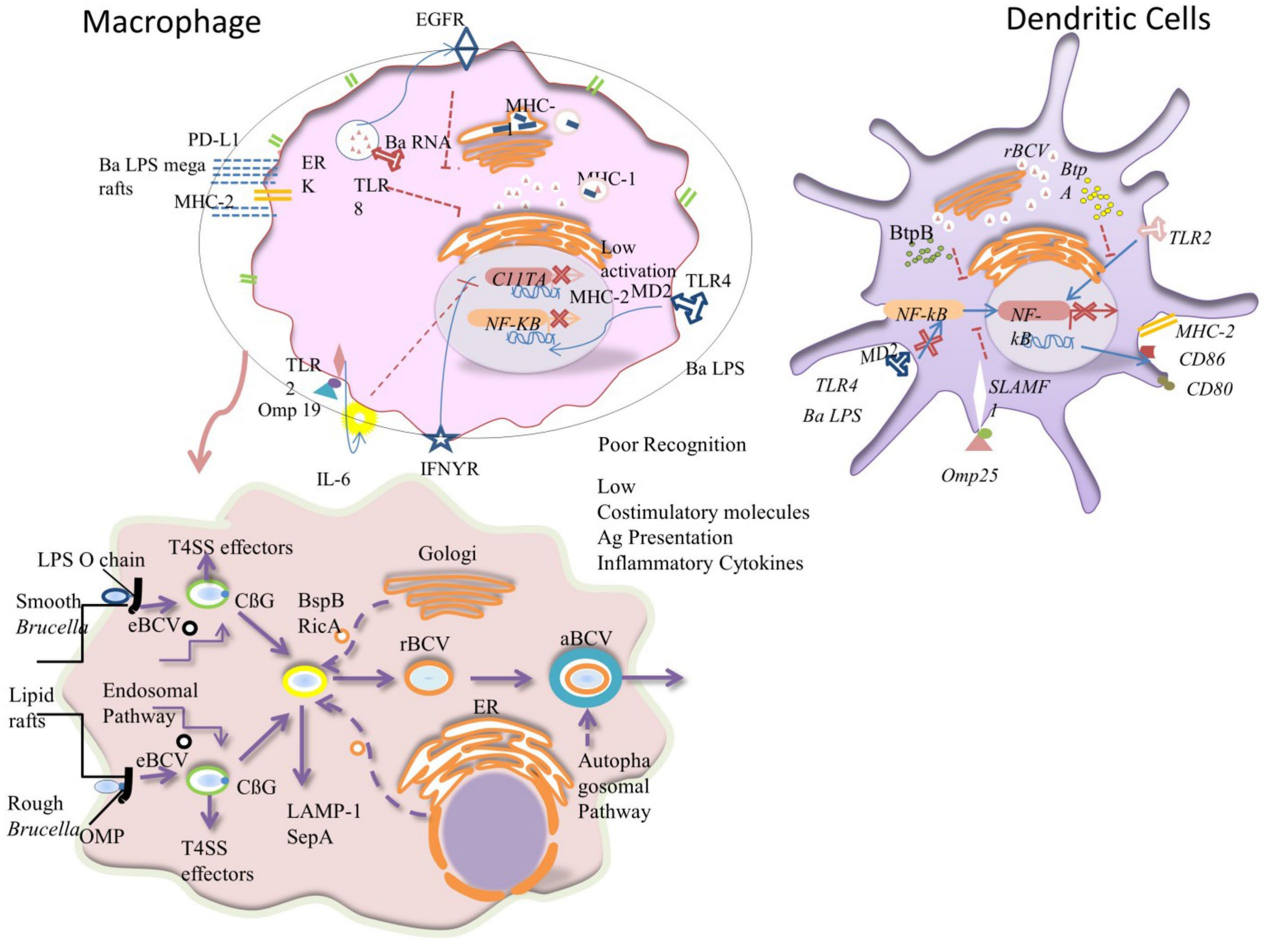
Mechanisms causing poor antigen presentation, intracellular trafficking, and penetration of *Brucella* inside macrophage: *Brucella* has developed a number of strategies to inhibit DCs’ and macrophages’ efficient antigen presentation, impairing the development of an adaptive immune system. (A) *Brucella* lipoprotein recognition by TLR2 results in IL-6-dependent suppression of the transcription factor CIITA, which reduces the transcription and expression of MHC-II that is triggered by IFN-γ. Additionally, *B. abortus* (Ba) LPS reaches the cell surface where it joins with MHC-II molecules to generate macrodomains that prevent peptides from being presented to CD4+ T cells. Since *B. abortus* RNA detection results in the retention of MHCI molecules within the Golgi apparatus via TLR8 and the EGFR pathway, this impairment also affects cytotoxic CD8+ T cells. Pro-inflammatory cytokine production and the expression of co-stimulatory molecules in DCs are decreased because of the Omp25-SLAMF1 interaction’s restriction of NF-kβ translocation to the nucleus. The maturation of DCs is regulated by the *Brucella* effectors BtpA and BtpB, which are translocated to the cytoplasm during infection and disrupt TLR2 and TLR4 signaling. Due to the Ba LPS’s unusual shape, particularly its core, it is poorly recognized by the TLR4-MD2 complex in both cell types, which prevents full activation, NF-kβ translocation, and inhibits DCs maturation and T-cell activation. Red represents nuclear phosphorylated active NF-β dimers. The T4SS effectors, the LPS O-chain, OMP22, OMP25d, and cyclic 1, 2-d-glucan (CβG) assist in the remodeling of the lipid-rich regions of the exterior vacuole that results in a fusion with lysosomes for bacterial replication. T4SS proteins in the cytosol of the host cell enable interaction with the endoplasmic reticulum that is converted into the replicative vacuole. The black and orange circles characterize membrane vesicle trafficking from the endolysosomal pathway, Golgi apparatus, and endoplasmic reticulum to the *Brucella*-containing vacuoles (BCVs). The alteration in the colors of the BCV membranes symbolizes their change in structure as they switch from eBCVs to rBCVs. The outer blue membrane of the aBCV shows the engulfment of the rBCV by the host cell autophagosomal pathway.

T4SS regulates the inflammatory response and manipulates vesicle trafficking inside host cells, thereby playing crucial roles in the inhibition of the host immune response and intracellular survival during infection. In brief, after the entrance of *Brucella* into the host cell, it lives in acidified phagosomal compartments that are recognized as (eBCVs), and the pH can reach 4, which is essential for the survival of *Brucella* and the intracellular expression of the T4SS. The eBCVs avoid fusion with the terminally degraded lysosomes, thus ensuring not only the intracellular survival of bacteria but also triggering intracellular bacterial growth before rBCV is formed (121). The rBCVs maintain *Brucella’s* chronic intracellular persistence in the host. On the other hand, aBCVs are thought to be crucial for bacterial infection and cell-to-cell spread in the host and are created when the rBCVs engage with elements of the host cell’s autophagy process (121). Overall, *Brucella* can resist the phagocytic bactericidal effect; and it can also induce the host cells to create a microenvironment that is favorable for bacterial survival, reproduction, and replication. This ability allows *Brucella* to remain in the host cells for extended periods, which ultimately results in the development of chronic persistent infection (113) (Figure 4).

### 4.3. Acquired immunity

**Figure 5:**
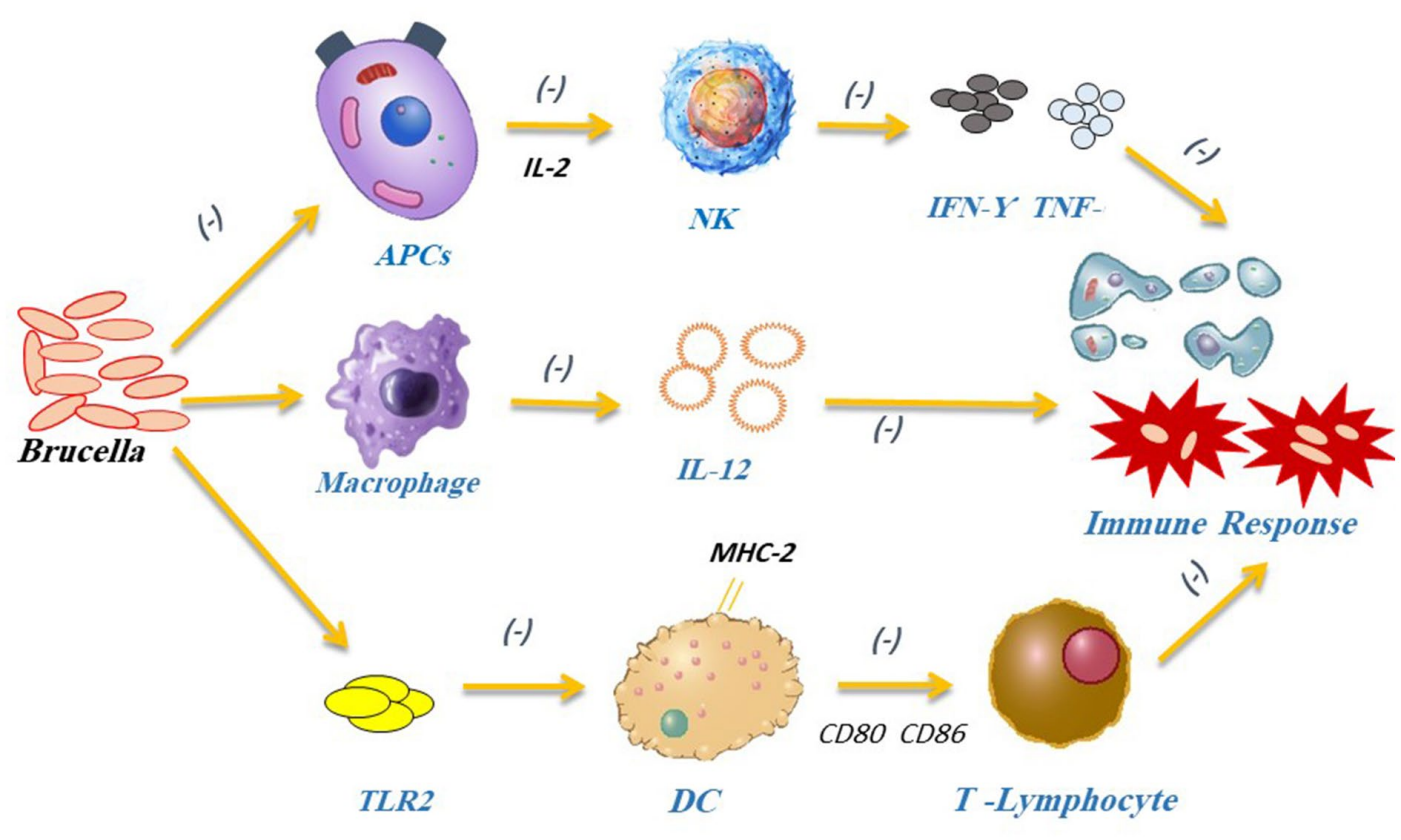
*Brucella* can stop APCs from secreting IL-2, which subsequently prevents the production of inflammatory substances such as IFN-γ and TNF-α by NK cells. *Brucella* can block IFN-γ-mediated phagocytosis to avoid being destroyed by the immune system. *Brucella* interferes with the Th1 immune response causing macrophages to lower IL-12 release and preventing DCs from activating T lymphocytes. It also impacts DCs maturation by blocking the TLR2 receptor pathway.

The other arm of host defense is acquired immunity. It consists of T lymphocytes, which are involved in cytokine production and cytotoxicity (cellular immunity), in addition to antibody-producing B lymphocytes (humoral immunity) (108). Primarily, the antigen-specific T cells secrete interferon-gamma (IFN-γ) as part of the Th1 immune response against *Brucella*. Then, the IFN-γ activates macrophage bactericidal machinery, enhances the production of antigen presenting cells (APCs), stimulates cytotoxic T-cells (CTL)-mediated cytotoxicity, and potentiates infected macrophage apoptosis (Figure 5). Finally, antibody-mediated opsonization (by IgG1, IgG2a, and IgG3) improves the phagocytic uptake of bacteria by lowering the initial level of *Brucella* infection (122). Studies showed that *Brucella* induces APCs to generate IL-2 and activates NK cells. These NK cells release TNF-α, IFN-γ, granulocyte-macrophage colony-stimulating factor (GM-CSF), and other cytokines that play a key role in Th1 and type 1 CD8+ T-cell (Tc1) responses (112). TNF-α can boost macrophage bactericidal ability. On the other side, IL-12 can activate the Th1 immune response and produce IFN- γ (123). IFN- γ secretion controls MHC-I and MHC-II expressions, which in turn, works for *Brucella* elimination through mediating Th1 immune response (111, 124). All in all, the decreased CD8+ T-lymphocyte activation, IL-12, and TNF-α lead to immunosuppression, and promote *Brucella* multiplication and infection persistence (125).

**Figure 6:**
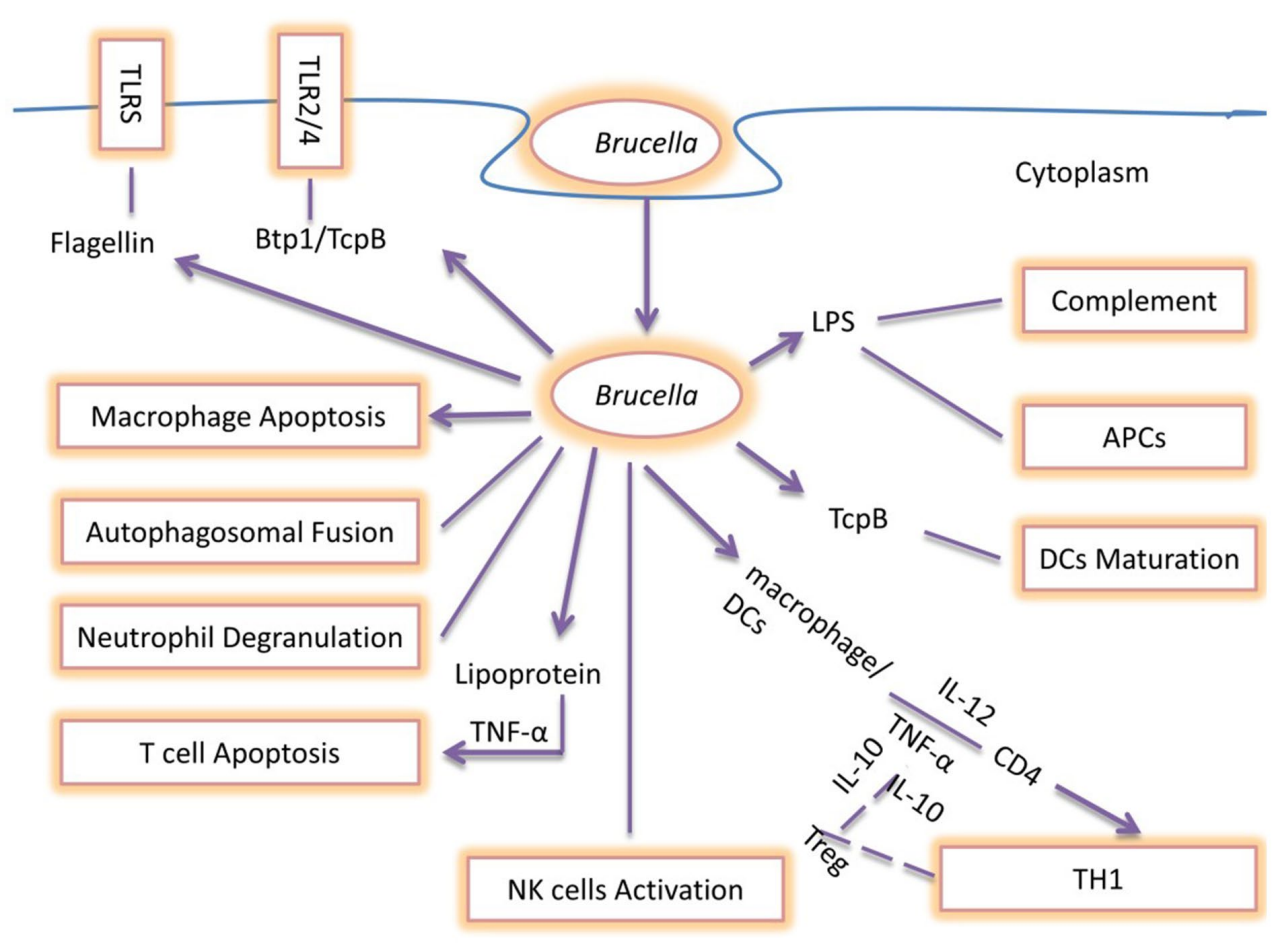
Summary of immune escaping Strategies of *Brucella*: During the early stages of infection, TNF-α and IL-12 are secreted by DCs and macrophage cells as a result of the detection of *Brucella*, which promotes intracellular survival and replication. Flagellin suppresses the TLR5 receptor, whereas *Brucella* produces the Btp1/TcpB protein, which blocks the TLR2/4 signaling pathway. The *Brucella* LPS O-antigen binds to C3, preventing the complement cascade from being activated. *Brucella* blocks neutrophil degranulation and NK cell activation, interfering with the host’s innate immune response. These bacteria produce Btp1/TcpB to prevent DCs maturation. *Brucella* LPS prevents antigen presentation and interferes with the host’s innate and adaptive defense, making chronic infection more likely. TNF-α secretion is necessary for *Brucella* lipoprotein to promote T-cell apoptosis, which in turn directly suppresses the T-cell response. Additionally, *Brucella* prevents macrophage apoptosis and autophagolysosomal fusion, which represents the primary evading mechanisms of *Brucella* pathogens. The end effect of interfering with these mechanisms is the development of human brucellosis and its clinical symptoms in natural hosts.

Recent studies clarified the roles of *Brucella* TIR protein 1 (Btp1), LPS, and PrpA as being significant immunomodulatory molecules with the capability to modify host immune mechanisms. Moreover, they can inhibit the secretion of IFN-γ and increase the secretion of IL-10 affecting Th1 immune response (126). Btp1 shows a sequence resembling the Toll/IL-1 receptors (TIRs) domain family. Different studies investigated the role of Btp1 in DCs maturation due to the significance of the TIR domain in TLR signaling. Btp1 inhibits both the production of proinflammatory cytokines and DCs maturation which leads to inhibition of TLR2 and TLR4 signaling. *Brucella* lumazine synthase (BLS) also induces negative effects by blocking the TLR4-myeloid differentiation protein-2 (MD2) complex and inducing CD8+ T-lymphocyte toxicity. This inhibition of CD8+ T-cell killing of *Brucella* target cells represents an adaptive immune evasion strategy (127). In addition, Btp1 binds to adapter TIRAP (TIR domain-containing adaptor protein) at the cell membrane and blocks NF-κβ activation (128). PrpA also plays a significant role in the early stages of *Brucella* infection by regulating IFN-γ, TNF-α, IL-10, and transforming growth factor-1 (TGF-1) pathways (129) (Figure 6).

#### 4.3.1. Vaccination

##### 4.3.1.1. Requirement of brucellosis vaccines

Although *Brucella* vaccines have been employed as a control measure, they are not 100% successful in preventing the disease (130). Average exposure to *Brucella* protects only 65% of vaccinated animals (131). The requirements for an optimal *Brucella* vaccination have remained unchanged since they were first established many years ago. Such a vaccine should be safe and effective, does not enhance antibodies interfering with serodiagnosis, cannot be transmitted to humans or other animals (including no contamination of milk, edible organs, dairy products, or meat), be stable *in vitro* and *in vivo*, be easily cultivable for large-scale production, and be supplemented with markers to be differentiated from the field isolates (29).

##### 4.3.1.2. Classic attenuated *Brucella* vaccines

*Brucella* S19, RB51, 45/20, and Rev1 strains are commonly used for designing vaccines to protect livestock against *Brucella* infections and subsequent abortions (132, 133) (Table 3). Natural attenuation of the erythritol catabolic genes in *B. abortus* S19 resulted in the loss of a 720-bp area compared to the primary strain which resulted in a less virulent strain (137). Vaccination with this attenuated strain stimulates a high level of immunity and protects animals against *Brucella* infection for a long period, which can extend to the entire productive life of the animal (138). On the other hand, the S19 vaccine has several drawbacks, including interfering with serodiagnostic brucellosis tests, inducing abortions in some vaccinated and pregnant animals, reducing milk production, and being pathogenic in humans (139). It is administered to female calves between the ages of 3 and 6 months in a single subcutaneous dosage of (2.5–12 × 10^10^) colony-forming units (CFUs) (140). Adult cattle can be injected with a lower dosage of organisms subcutaneously, but some animals will acquire persisting antibody titers and may abort and excrete the vaccine strain in their milk. When used for calves aged 3–5 months, the conjunctival technique is completely safe and minimizes the risk of serological interference. It can also be given to cattle of any age as one or two doses of 5 × 10^9^ CFU via the conjunctival route, which protects without a prolonged antibody response and minimizes the risk of abortion and microbial shedding in milk when vaccinating adult cattle (141). Subsequently, Saidu et al. confirmed that the intraocular route is still the safest route for vaccinating adult cattle than the subcutaneous route (134).

**Table 3:** The advantages and disadvantages of the classic attenuated *Brucella* vaccines.

Vaccines	Advantages	Disadvantages	Notes	References
Smooth *B. abortus* S19	Efficacy in control/eradication programs has been demonstrated (USA, EU countries, and Australia). It provides superior protection for cattle against *B. abortus* and *B. melitensis* A single dose provides effective protection for the rest of life.	When used in pregnant cattle, depending on the dose and immunization route, it can cause abortion. It is not safe in bulls when used subcutaneously (unknown safety when applied by the conjunctival route). Serological interference with (RBT, CFT), iELISA and cELISA, fluorescence polarization assay, and other S-LPS tests. It exhibits substantial human virulence	When young animals are given lower doses, serological interference is reduced (particularly by the conjunctival route) Conventional serological testing can be used to diagnose human infections, and standard antibiotic treatment can be used to treat them.	(29, 134)
*B. melitensis* Rev. 1	Efficacy in control/eradication programs has been demonstrated (France, Italy). Both *B. melitensis* and *B. ovis* are susceptible to it. Males and young replacements are safe. A single dose provides effective protection for the rest of life.	Abortion is at a high level. Serological interference with RBT, CFT, indirect and competitive ELISA, fluorescence polarization assay, and other S-LPS tests. It exhibits substantial human virulence, and it is resistant to streptomycin.	By avoiding the vaccination of pregnant animals *via* the conjunctival route, safety concerns are reduced. When given to young animals *via* the conjunctival route, serological interference is reduced. Standard serological tests can be used to diagnose human infections; however, treatment strategies that do not use streptomycin are required.	(135)
Rough *B. abortus* RB51	When young animals are given lower doses, serological interference is reduced (particularly by the conjunctival route). Conventional serological testing can be used to diagnose human infections, and standard antibiotic treatment can be used to treat them.	It causes fewer abortions in cattle compared to the S19 vaccine. Inducing protective immunity is less successful than S19 (efficacy or revaccination unknown) The protection duration is unknown. Cattle protection against *B. melitensis* is unclear. Indirect and competitive ELISAs, as well as fluorescence polarization assays, are all affected by serological interference. It is resistant to rifampicin.	It is currently licensed in non-pregnant female cattle (4–12) months. There is no evidence of eradication efficacy. There are no adequate serological diagnostic tests for human infections, and therapy needs non-rifampicin regimens. It has been documented to cause human disease, most commonly through occupational exposures such as needle sticks.	(29, 136)

*B. abortus* strain RB51, a rifampicin-resistant mutant, was isolated as a single rough colony from a virulent smooth strain of *B. abortus* 2,308. Serial subculturing of *B. abortus* 2,308 resulted in the deletion of the wbo A gene, which encodes a glycosyltransferase required for O-side chain production (29, 142). Unlike the strain 19 vaccine, the strain RB51 vaccine does not affect *Brucella* serodiagnosis results (143). Therefore, it has replaced the *B. abortus* strain 19 vaccine, which is well-known as the calf hood vaccine in many countries. The term calfhood vaccine means vaccination of heifers between 4 and 10 months of age with *Brucella abortus* strain 19, and the best age of vaccination is at 5 months (144). RB51 vaccine can infect humans, and one of its primary drawbacks is the resistance to rifampicin antibiotic, which is used for human brucellosis therapy (145). *B. melitensis* Rev1 vaccine could be used to protect sheep and goats against brucellosis (146). However, it has a negative effect because it might cause abortions in pregnant animals after vaccination (24). Aside from being virulent for humans, lactating female animals can shed the vaccinal strain through milk, which can infect other animals and interfere with serological diagnosis (147).

*B. abortus* 45/20 strain was created by passing the *B. abortus* 45/0 virulent strain 20 times in guinea pigs, although multiple studies have shown that it can revert to smooth virulent forms when used as a live vaccine. So far, the 45/20 vaccine is combined with an oil-based adjuvant, needing repeat vaccination (143). A systematic review and meta-analysis were performed to recalculate the vaccine efficacy of *B. abortus* S19 and RB51 vaccine strains suggesting that a dose of 10^9^ CFU for S19 and 10^10^ CFU for RB51 are the most effective for the prevention of abortion and infection caused by *B. abortus* (136).

The DIVA vaccines (Differentiating Infected from Vaccinated Animals) are defined as the vaccines that distinguish infected and vaccinated animals. S19 and Rev1 vaccine problems can be solved using DIVA vaccines. There are two main strategies to produce these vaccines. First, the removal of the diagnostic antigens found in field strains from current vaccines. In other words, creating *Brucella* vaccine candidates free from O-chain and NH-polysaccharides or synthesis of immunogenic proteins using *Brucella* mutants that could be used as neutral antigenic markers. Second, the addition of the foreign (xenogenic) antigens to the classical live-attenuated *Brucella* vaccine strains, thereby enabling the development of diagnostic tests that can recognize vaccinated animals in infected environments (141). Meanwhile, DIVA vaccines that can replace S19 and Rev1 vaccines should protect at least as good as the primary vaccines (29). Many studies have suggested several vaccine candidates for DIVA vaccines such as VjbR and BtpA proteins contributed to the virulence of the Y3 and M5 *Brucella* strains. Zur protein, ABC transporters, and thiamine metabolism-associated proteins may play crucial roles in *Brucella* survival and pathogenesis (148). In parallel, Uslu and Erganis revealed that *B. melitensis* Rev.1 ΔOmp19 can act as a DIVA marker vaccine using an ELISA test for the detection of the Omp19 protein (149).

##### 4.3.1.3. Genomics and proteomics approaches for improved *Brucella* vaccines

###### 4.3.1.3.1. *Brucella* subunit vaccines

Several *Brucella* fragments, such as recombinant peptides, proteins, DNA, LPS, and outer membrane proteins (OMPs) are now being tested as subunit vaccines against *B. abortus* (147). They have been proved to be a promising field for study and development because of their pioneer advantages over traditional live-attenuated vaccines, including high safety with no residual virulence and the ability to be used in people and pregnant animals. However, although they provide appealing alternatives to traditional live-attenuated vaccines, they face significant challenges. The low protective efficacy and the requirement for adjuvant and booster doses are among them. Consequently, subunit vaccines can be improved by using potent T-cell antigens that produce a Th1 immune response as dominant immunity against brucellosis (150, 151) (Table 4).

**Table 4:** The immune response of *Brucella* subunit vaccine candidates.

Subunit vaccine candidates	Immune response	References
LPS and OMVs	However, because of their high safety without residual virulence and possible use in humans and pregnant animals, they have low protective efficacy and need for adjuvants and booster shots.	(152–154)
OMP16, OMP19, liposomes protein L7/L12, OMP25, p39, and AsnC are *Brucella* subunit proteins	Stimulate Th1 immunity and protect that provided by the commercial S19 live vaccine.	(152–154)
Dihydrolipoamide succinyltransferase (rE2o) and cysteine synthase A (rCysK) subunit vaccines	Th2 immunity was evoked	(155, 156)
Cytosolic proteins SurA and DnaK	Elicits lesser levels of protection against *B. abortus* in mice.	(157)
Recombinant protein cocktail (rOMP19 and rp39)	Th1-mediated isotype antibodies and cellular immunity	(158, 159)
*Brucella* OMP25c recombinant protein combined with Freund’s adjuvant produced	Produces both Th1 and Th2 immune responses in mice, with protection levels comparable to the S19 strain.	(160)
AspC, Dps, Ndk, and lnpB as *B. abortus* proteins subunit vaccines	Elicits high levels of IgG2a titer and exhibited equal protective efficacy to the RB51 strain.	(161)
3E-IL2 *Brucella* recombinant vaccines	Stimulate the immune system to create Th1 cytokines and antibodies. Generate larger amounts of IFN-γ and IL-2.	(162)
DNA vaccines encoding BAB1-0263 and BAB1-0278 from ORFs of GI-3	Stimulate both cellular and humeral immunity with a high level of IF-γ. DNA vaccines express BAB1-0278 protected mice when challenged with *B. abortus* 2,308 strain	(137) (144)

OMP16, OMP19, liposomes protein L7/L12, OMP25, p39 (a potential periplasmic binding protein), and AsnC are important *Brucella* subunit proteins (152–154). In general, these antigens stimulate Th1 immunity and provide protection similar to that provided by the commercial S19 live vaccine (153, 158). Other *Brucella* subunit vaccines such as dihydrolipoamide succinyltransferase (rE2o) and cysteine synthase A (rCysK) are capable of inducing Th2 immunity with a relatively low protection level (155, 156). In addition, compared to the S19 vaccine, the SurA and DnaK cytosolic proteins elicit lower levels of protection in a mouse model (157). Additionally, *B. abortus* rAdk and rSecB have been suggested as promising subunit vaccine candidates. BP26 and BLS proteins are also among the top candidates for serological diagnosis of brucellosis (154, 163).

Vaccinating mice with a recombinant protein cocktail (rOMP19 + rp39) resulted in Th1-mediated isotype antibodies and cellular immunity, which protected animals against the *B. abortus* 544 strain (158). Afterward, the *B. abortus* chimeric subunit protein with OMP19 and p39 domains was used as a booster dose-induced Th1-type immune response (159). Intriguingly, using *Brucella* OMP25c recombinant protein combined with Freund’s adjuvant produced both Th1 and Th2 immune responses in mice, with protection levels similar to the S19 vaccine (160). More interestingly, using a mixture of various recombinant *B. abortus* proteins, including AspC, Dps, Ndk, and lnpB as subunit vaccines, induced high levels of IgG2a titer and exhibited equal protective efficacy to the RB51 vaccine strain (161). In comparison with other recombinant subunit proteins, 3E-IL2 inoculation could be a superior candidate for further study into the manufacture of *Brucella* recombinant vaccines (162).

###### 4.3.1.3.2. DNA vaccines

Another type of subunit vaccine that induces strong humoral and cellular immune responses with repeated doses is the DNA-based *Brucella* vaccine (164). Using laboratory and clinical testing, they were proven to be safe and effective. To examine their immunogenicity, BALB/c mice were immunized with multivalent DNA vaccines. These vaccines significantly induced the humoral immune response (IgM, IgG, and IgG2a) and cell-mediated immunity (high IFN-γ and increased splenic lymphoproliferative response) (165). The genomic island 3 (GI-3) region of *B. abortus* encodes multiple open reading frames (ORFs) that express critical antigens for microbial intracellular survival and pathogenicity. Therefore, the *Brucella* DNA vaccine based on the GI-3 region could be a good candidate for protection against *B. abortus* infection (166). More specifically, DNA vaccines expressing BAB1-0263 or BAB1-0278 genes from GI-3 ORFs induced substantial levels of IFN-γ production, which in turn elicited strong humoral and cellular immunity. In addition, when mice were challenged with the *B. abortus* 2,308 strain, a DNA vaccine expressing BAB1-0278 provided good protection (167).

In comparison with *B. abortus* RB51 vaccination, DNA vaccine encoding Cu-Zn superoxide dismutase (SOD)-IL-2 fusion protein stimulated IgG2a and TNF-α in mice, resulting in excellent protection against *B. abortus* 2,308 strain (165). The combination of SOD with L7/L12 and BCSP31 generated a robust cytotoxic CD8+ T cell and specific IgG, resulting in a higher level of protection compared to *B. abortus* S19 (168). In another investigation, *Brucella* genes (SOD, BCSP31, and L7/L12) were joined with several genes from *Mycobacterium bovis* or *Mycobacterium TB,* resulting in a dual efficient DNA vaccine for both infections (*Brucella and Mycobacteria*) (169, 170). Another study showed that a divalent DNA vaccine expressing both *B. abortus* L7/L12 and OMP16 genes boosted robust cellular and humoral immunity in mice by inducing IFN-γ and IgG2a production. In addition, compared to the univalent OMP16 or L7/L12 DNA vaccine, this divalent DNA vaccine induced higher levels of protection, even though the protective efficacy of the divalent OMP16 and L7/L12 was lower than that of the traditional RB51 vaccine (171).

The bacterial ghost (BG) vaccine is a novel strategy with great promise. In China, the *B. abortus* A19 strain is widely used as a vaccine. However, persistent pathogenicity in animals and humans is a common disadvantage (172). In China, He et al. developed the *Brucella abortus* A19 bacterial ghost (A19BG) vaccine; it was inactivated using a two-step process that included biological lysis and hydrogen peroxide treatment, which, in turn, completely inactivated the viable bacterial cells found even at high concentration of 10^10^ CFU/mL. Furthermore, no adverse effects have been noticed in guinea pigs. The levels of antibodies, interleukin-4, interferon-γ, and CD4+ T cells in guinea pigs inoculated with the A19BG vaccine were comparable to those inoculated with the conventional A19 vaccine. In guinea pigs and cattle, vaccination with both A19BG and A19 vaccines provided equal protection against *B. melitensis* M28 (173).

###### 4.3.1.3.3. Genetically engineered live-attenuated vaccines

The identification of genes linked to virulence or organism survival can help in creating safe and efficient novel vaccines. In comparison with traditional live-attenuated vaccines, currently developed live-attenuated vaccines can induce significant levels of protection (137). The purine biosynthesis pathway genes, T4SS, virB genes, lipid A fatty acid transporting gene, ferrochelatase hem H mutant, phosphoglycerate kinase encoding gene, and the LPS biosynthesis pathway gene are considered under vaccinal development depending on multiple deletions in *B. abortus* genome that lead to significant attenuation (174, 175). These mutations provide supreme protection compared to traditional live-attenuated vaccines. In mouse and human cell experiments, deletion of *B. abortus* 2,308 norD and high-affinity zinc uptake system (znuA) genes causes adequate attenuation. Furthermore, this live recombinant strain effectively enhances T cells and produces potent immunity against virulent challenges, compared to traditional RB51 vaccinated groups (176).

By deleting the phosphoglucomutase (pgm) gene of *B. abortus* 2,308, Ugalde et al. created a recombinant strain with no serodiagnostic interference and protective Th1 immune responses compared to the S19 strain (177). Additionally, GntR, a transcriptional regulator of many virulent antigens of *B. abortus* 2,308, has been deleted resulting in an attenuated mutant with high protection levels in mice against the parental *B. abortus* 2,308 challenge (178). In parallel, the deletion of NodV and NodW genes in *B. abortus* 2,308 resulted in an attenuated live vaccine with reduced survival in cell lines and mouse models, and it did not affect serological diagnosis (179). In 2010, Yang et al. successfully deleted znuA and purE genes of *B. abortus* resulting in a live-attenuated mutant that required two doses to generate an appropriate immune response in mice (180).

The deletion of the cgs gene in *B. abortus* S19 increased the attenuation of the S19 strain without altering its protective efficacy against *B. abortus* 2,308 (181). In addition, the deletion of the vjbR gene, which is responsible for intracellular *Brucella* persistence, resulted in a recombinant mutant with high levels of protection and reduced inflammation (182). *B. abortus* targeted mutants with deletions in the membrane fusogenic protein (Mfp) or OMP19 genes decrease *Brucella* persistence in animal studies. However, challenge tests have revealed that traditional attenuated vaccines such as S19 and RB51 strains provide similar levels of protection (183). The deletion of the wbkC gene of *B. abortus* (ΔwbkC), which translated to formyltransferase enzyme, showed a protection level similar to *B. abortus* rough strain RB51 avoiding the rifampicin resistance which is the main disadvantage of RB51. However, *B. abortus* ΔwbkC did not produce the same level of protectin when compared to *B. abortus* S19 and avoided interfering with the serological diagnosis which is also the main disadvantage of the S19 vaccine strain (184). The Glycosyltransferase Wad C gene is involved in the production of the core oligosaccharide region of *B. abortus* LPS and is essential for efficient innate immune recognition (185). The deletion of this gene produced a better immune response compared to those provided by the S19 strain (186).

Single and double gene deletions of the virulent *B. abortus* RB51 cydD and cydC genes have created significantly attenuated mutants (RB51ΔcydC, RB51ΔcydD, RB51ΔpurD, RB51ΔcydCΔcydD, and RB51ΔcydCΔpurD). Vaccination with a single dose of these mutants induced lower protective immunity in mice compared to the RB51 vaccine. However, a booster dose provided significant levels of protection in mice against the challenge of the virulent *B. abortus* strain 2,308 and *B. canis* strain 26 (145, 187). Additionally, in South Korea, the double deletion in a field isolate of *B. abortus* biovar 1 (BA15) produced similar protective effects without the need for a subsequent booster shot (188). In *B. abortus* biovar 1 strain IVKB9007, deletion of the ATP/GDP-binding protein motif A (p-loop) and the ATP-binding/permease protein resulted in attenuated mutants that were unable to replicate intracellularly in cell line models. These mutants induced significant levels of protection against the pathogenic *B. abortus* strain 544 (189). Another study compared the whole-genome sequences of *B. abortus* S19 and A19 vaccine strains. Both strains were proved to have a significant degree of genetic similarity. The differences in genomic structure revealed that S19 had a 697 bp deletion and loss of function on both eryC and eryD genes compared to the A19 strain (190). Moreover, the Wzm/Wzt system has an important role in exporting the O-polysaccharide (O-PS) as the main virulence factor of *Brucella*. Single and double deletion mutants of wzm/wzt have been designed using the attenuated strain *B. melitensis* Rev1. Results revealed that Rev1Δwzm mutant is an immunogenic and effective vaccine candidate against *B. melitensis* and *B. ovis* in mice even though it had low persistence. So, it has minimal serological interference in sheep making Rev1Δwzm a highly promising vaccine candidate. Rev1Δwzm is a highly promising vaccine candidate in sheep because it has minimal serological interference and is safe to be used in pregnant ewes (191) (Table 5).

**Table 5:** The genetic engineering *Brucella* vaccines.

Genetic engineering vaccines	Significance	References
Deletion of the *B. abortus* 2,308 norD and high-affinity zinc uptake system (znuA) genes	Enhanced T cells and pro-inflammatory cytokines, compared to traditional RB51 vaccinated groups.	(176)
Deleting the phosphoglucomutase (pgm) gene of *B. abortus* 2,308	Produced a recombinant strain with no serodiagnostic interference and protective Th1 immune responses compared to the S19 strain.	(177)
Deletion of GntR, a transcriptional regulator of many virulent antigens in *B. abortus* 2,308	Resulted in an attenuated mutant with high protection levels in mice against the parental *B. abortus* 2,308 challenge	(178)
Deletion of the NodV and NodW genes in *B. abortus* 2,308	Lowered survival in cell lines and a mouse model, as such it does not affect serological diagnosis.	(179)
Deleted znuA and purE genes in *B. abortus* 2,308	Resulted in a live-attenuated mutant that required two doses to generate appropriate immune responses in mice.	(180)
Deleting the cgs gene in *B. abortus* S19	Increased attenuation of S19 strain without altering its protective efficacy against *B. abortus* 2,308	(181)
Deletion of the vjbR gene in *B. abortus* S19	Resulted in a recombinant mutant with higher levels of protection, reduced inflammation, and safety than S19.	(182)
Deletion of the membrane fusogenic protein (Mfp) or OMP19 genes	Lowered *Brucella* persistence in animal studies. However, challenge tests have revealed that traditional attenuated vaccines such as S19 and RB51 strains provide similar levels of protection	(183)
Deletion of the wbkC gene of *B. abortus* (ΔwbkC) which translated to formyltransferase enzyme	Showed a protection level similar to *B. abortus* rough strain RB51 avoiding the rifampicin resistance which is the main disadvantage of RB51. However, *B. abortus* ΔwbkC did not produce the same level of protection when compared to *B. abortus* smooth strain S19, and avoiding interfering with the serological diagnosis which is also the main disadvantage of the S19 vaccine strain	(184)
Glycosyltransferase Wad C gene deletion in *B. abortus* S19	Produced a better immune response comparable to those provided by the S19 strain	(186)
Single and double deletions of CydC cydD and CydC purD genes in *B. abortus* RB51	Produced substantial attenuated mutants in cell lines. Additionally, compared to the RB51 strain, mice examination showed a Th1-type immune response and strong protective efficiency against *B. abortus* 2,308 strain infection	(145) (187)
Double deletion of CydC cydD and CydC purD genes in a field isolate of *B. abortus* biovar 1 (BA15)	Produced similar protective effects without the need for a subsequent booster shot	(188)
Deletion of the ATP/GDP-binding protein motif A (p-loop) and the ATP-binding/permease protein (cyd C) in *B. abortus* biovar 1 strain IVKB 9007	Resulted in effective attenuated mutants that were unable to replicate intracellularly in a cell line model	(189)
Single and double deletions of wzm/wzt using the attenuated strain *Brucella melitensis* Rev1	It has minimal serological interference asd it is not causing abortion in pregnant ewes.	(191)

###### 4.3.1.3.4. Vector-based *B. abortus* vaccines

Several *Brucella* vaccines based on bacterial or viral vectors have recently been designed, and they provide an efficient method of delivering a range of heterologous or homologous antigens (192) (Table 6). The optimum method for stimulating the target host immune system against *Brucella* pathogens can be achieved through induced cell-mediated immunity because of the intracellular tropism of this microorganism. These live vaccines produce many copies of the *Brucella* antigens because they reproduce inside the host cells. Despite all these benefits being highly appealing, there is no current efficient vector-based *Brucella* vaccine that provides the best protection.

**Table 6:** The vector-based *B. abortus* vaccines.

Vector-based *B. abortus* vaccines	Significance	References
A combination of *Salmonella* vectors expressing BCSP31, SOD, and OMP3b	Increased Th1 response and enhanced protection	(193)
*Salmonella* as a vector that expressed ribosomal protein L7/L12 and the lumazine synthase enzyme	Induced a Th1-type response but did not protect mice against *B. abortus* challenge.	(194, 195)
Attenuated *Salmonella* vector-based vaccine that expressed heterologous *Brucella* antigens (SOD, BLS, PrpA, and OMP19)	Produced significant levels of IFN-γ and provided adequate protective efficacy that was alternative to the traditional RB51 vaccine.	(196)
Influenza viral vector-*B. abortus* vaccine	Provided equal protection to the *B. abortus* S19 vaccine.	(197)
Adenovirus vector-based vaccine that expressed the p39 and lumazine synthase proteins of *B. abortus* for immunization of mice	Induced substantial humoral and cellular immune responses in a mouse model.	(198)

Many studies demonstrated that attenuated Salmonella strains can express various *Brucella* antigens, such as BLS, PrpA, Omp19, and Cu-Zn SOD, which were employed as vaccine vectors. These formulations with pure *Brucella* LPS were inoculated intraperitoneally in specific pathogen-free (SPF) mice and produced an efficient delivery with a significant (*p* ≤ 0.05) reduction in splenic wild-type *B. abortus* 544 colonization compared to the control mice. More importantly, this vaccination system can provide a platform for the development of cross-protecting vaccines able to prevent multi-species brucellosis (199).

As vector-based vaccines, influenza viruses expressing *Brucella* ribosomal proteins L7/L12 and OMP16 have been produced (200). Cross-protection against *B. melitensis* can be obtained using the *B. abortus* vaccine. First, pregnant heifers received an influenza viral vector *B. abortus* vaccine and have been protected from abortions. After challenging with *B. melitensis*16 M, a remarkable cross-protection (90–100%) was obtained for the heifers, their calves, and fetuses. On the other hand, when compared to the *B. abortus* S19 vaccine, the influenza viral vector-*B. abortus* vaccine provided equal protection (197). In terms of safety, this recombinant vector vaccine showed superior safety characters. Notably, this vaccine prime-booster immunization produced humoral and cellular immunity with long-term protection against *B. abortus* infection, particularly in pregnant heifers (201, 202). In addition, adding OMP19 and SOD proteins to the vaccine formulation, as well as using montanide gel as an adjuvant, resulted in excellent protection in sheep and goats when challenged with *B. melitensis* (203).

An adenovirus vector-based vaccine was developed for protection against brucellosis and expressed the p39 and BLS proteins of *B. abortus*. This vaccine successfully induced strong humoral and cellular immune responses in a mouse model (198).

###### 4.3.1.3.5. Proteomics approaches

Proteomics, the key post-genomic technique for analyzing the effects of regulatory mechanisms on the protein composition of microorganisms, has the potential to deal with issues of One-Health concern. It is a useful tool for researching gene expression, microbial physiology, and host–pathogen interactions. Scientists have been using proteomics technology to decipher numerous mysterious areas of *Brucella* research since the start of the 21st century. Proteomics techniques, including sodium dodecyl sulfate-polyacrylamide gel electrophoresis (SDS-PAGE), two-dimensional gel electrophoresis (2DE), matrix-assisted laser desorption ionization–time of flight mass spectrometry (MALDI-TOF MS), and label-free analysis, are advanced approaches for accurate analysis of pathogenic protein spots (204).

A study was performed to identify the phenotypic and pathophysiological variations among *Brucella* species (*B. melitensis* strain 16 M and *B. ovis* REO198) and to correlate these variations with virulence elements. SDS-PAGE was used to separate protein extracts from the two *Brucella* species. The nano-scale liquid chromatographic tandem mass spectrometry (nLC–MS/MS) was used to investigate the areas that were qualitatively and quantitatively different. A total of 274 and 606 proteins were identified for *B. melitensis* and *B. ovis*, respectively. Bioinformatics study was performed to examine their structural and functional characteristics. Outer membrane immunogenic protein N8LTS7 and outer membrane immunogenic protein Q45321 were identified as promising candidates for improving *Brucella* diagnostic procedures and vaccinations for *B. ovis* and *B. melitensis*, respectively (205). Moreover, outer membrane vesicles (OMVs) have been suggested as a paradigm for the development of cellular vaccines because they contain immunogenic proteins. To characterize the pan-proteome of these vesicles, OMVs of *B. suis*, *B. ovis*, *B. canis*, and *B. neotomae* were isolated and analyzed using SDS-PAGE, transmission electron microscopy, and liquid chromatography-mass spectrometry (LC–MS). Western blot was also used to identify antigenic proteins. Several homologous immunogenic proteins, including Omp25, Omp16, Omp2a, Omp31, SodC, and BhuA, were detected during *in silico* investigations. Anti-*Brucella* sera were able to identify the proteins in the vesicles from several *Brucella* species (206).

A comparative study between the vaccinal strain (*B. melitensis* Rev.1) and the field strain (*B. melitensis* 16 M) enabled us to get the detailed *Brucella* protein repertoires using label-free shotgun proteomics investigation which would help in discriminating the vaccinated from infected animals (207). MALDI-TOF MS, a quick, affordable, and trustworthy way for routinely identifying Brucellae, is an advanced technique used to reduce the time needed to identify pathogens and may soon replace the current conventional and molecular procedures. Hamidi et al. performed the MALDI-TOF MS technique for fast and trusty differentiation of *B. abortus* and *B. melitensis*, depending on proteomic mass patterns. They reported significant protein mass signals, which were mapped to ribosomal proteins and structural proteins, such as integration host factor subunit alpha, GNAT family N-acetyltransferase, HU family DNA-binding protein, cold-shock proteins, and ATP synthase subunit C. They used these specific biomarker peaks that have been recognized for virulent and vaccine strains as differentiating tools (208). Furthermore, a unique MALDI-TOF MS reference library was created by Christoforidou et al., and the effectiveness of its performance on species-level identification was assessed using 75 *Brucella* spp. isolates. Analysis of mass peak profiles allowed for 100% accurate genus and species identification of *Brucella*. Despite the significant intrageneric similarity, the MALDI-TOF MS database was able to classify 47 out of 62 *B. melitensis* bv. 3 isolates at the biovar level (75.81%) (209).

Proteins and genes are differently expressed in *B. abortus* cultured under biofilm and planktonic conditions. 2DE and MALDI-TOF MS were used to separate and identify these proteins, respectively. 2DE revealed 20 protein spots that were differentially expressed between biofilms and planktonic cells, which matched 18 distinct proteins, including enolase and the elongation factor Tu. According to an RT-qPCR study, all 18 genes were downregulated in biofilms. Fourteen function and pathway-associated genes were reported using high-throughput sequencing and bioinformatics analysis. The 14 genes were upregulated in biofilm conditions and they may play important roles in bacterial defense, colonization, invasion, and virulence (210). A comparative *in silico* study comparing the proteomes of *B. melitensis* Rev.1 and 16 M was performed to identify biomarkers proteins, which distinguish the vaccine and the field strain. MALDI-TOF MS has identified two unique biomarkers most similar to ribosomal proteins (L24 and S12) (211). For accurate identification of *Brucella* species in animals and humans, Elbehiry et al. have recognized the proteomics of *Brucella* species with the help of the MALDI-Biotyper (MBT) system and then confirmed the results by microfluidic electrophoresis. This strategy was proved to be effective for the diagnosis of *Brucella* pathogens in animals and humans. Eleven positive *B. melitensis* isolates and 14 positive *B. abortus* isolates were reported (212).

Furthermore, Mahmud et al. studied the entire proteome of *B. abortus* strain 2,308 by using subtractive genomic analysis, and the results showed that only three membrane proteins (acriflavine resistance protein B, ABC transporter permease, and penicillin-binding protein 2) were observed to be potential novel vaccine candidates in cattle, while ABC transporter permease was predicted as a novel drug target for humans (213). *B. abortus* and *B. melitensis* outbreak strains from cows and sheep, respectively, were subjected to label-free quantitative proteomic analysis, which identified 402 differentially expressed proteins. Among these, 63 and 103 proteins were only found in the whole-cell extracts of *B. melitensis* and *B. abortus* field strains, respectively (214). Following numerous environmental pressures and adaptations, 1,221 differently expressed proteins in *B. abortus* were reported using label-free relative quantitative proteomics and mass spectrometry (215).

To investigate protein biomarkers for *Brucella* in human and livestock serum, a targeted multiple reactions monitoring-mass spectrometry (MRM-MS) method was created. In total, 30 synthetic peptides that match 10 immunodominant *B. abortus* proteins were created using bioinformatics analysis; and 117 serum samples from people and animals, classified as clinically confirmed (45), suspected (62), and control (10), were used to optimize the MRM-MS technique for the precise detection of these peptides. Transitions for four peptides were found in various clinically confirmed and suspected human and cattle serum samples using high-throughput MRM tests. Of them, peptide NAIYDVVTR with 100% specificity was repeatedly found in the clinically proved serum samples of both people and livestock, and it corresponds to the *B. abortus* protein (BruAb2_0537) (216).

With vaccine antigen prediction based on bioinformatics analysis of pathogen genomes, reverse vaccinology (RV) has been demonstrated to be a highly successful method (217). Therefore, based on the *Brucella* protein-coding genome, RV has been utilized in several research projects to evaluate prospective vaccine candidates (218). Zai et al. carried out a wide-scale study to screen putative antigens against 213 pathogenic strains of *Brucella* spp. with global geographic distribution using core proteome analysis and a compositive RV technique. Six biological characteristics (subcellular localization, antigen similarity, antigenicity, mature epitope density, pathogenicity, and adhesion probability) were used to rank candidate proteins. A total of 32 potential antigens were chosen for the RV analysis. Omp19 was used as a positive control, and T4SS protein VirB8 and type I secretion system (T1SS) protein HlyD were chosen for the evaluation of immunogenicity and preliminary protection in a mouse model (219). In addition, Vishnu et al. revealed eight proteins as potential vaccine candidates after thoroughly screening the exoproteome and secretome of *B. melitensis* 16 M. These proteins include the LPS-assembly protein LptD, heme transporter BhuA, hemagglutinin, flagellin FliC, 7-alpha-hydroxysteroid dehydrogenase, and immunoglobulin-binding protein EIBE. The functions of BhuA and hemagglutinin are crucial for the establishment of infection (220).

Moreover, Aslam et al. obtained the core genes set from 56 full genome sequences of *B. melitensis*. To find prospective proteins as drug targets and multi-epitope vaccine constructions from core genes, strict bioinformatics architecture of comparative genomics and RV techniques were used. Based on their function and specific metabolic pathways, lack of homology to human and human gut microbiome proteins, and potential for acting as drug targets, the 23 proteins were predicted as novel drug targets (221). Furthermore, apolipoprotein N-acyltransferase (Int) was found to be the probable target of the most abundant short RNA released during early infection with bone marrow-derived macrophages (BMDMs) in *B. abortus*. Int expression was shown to be reduced during BMDM infection, and immunoinformatic analysis of the protein sequence allowed the reasonable selection of a potential immunogenic epitope that was investigated as a vaccine candidate (222).

Moreover, a multi-epitope vaccine (MEV) was designed using the RV technique of Omp25, Omp10, Omp31, and BtpB for creating MEV. In addition, 9 linear B-cell epitopes, 2 conformational B-cell epitopes, 13 cytotoxic T-lymphocyte (CTL) epitopes, and 17 helper T-lymphocyte (HTL) epitopes were predicted and proposed for further research. To increase immunogenicity, the vaccine peptide’s N-terminal was supplemented with the proper adjuvants. A final MEV with 806 amino acids was created using linkers and adjuvants. The outcomes of the trials on animals showed that the MEV had a significant immunogenicity and could be used as a *Brucella* vaccine (223). Small extracellular vesicles known as exosomes are released by cells and serve as a means of intercellular communication. Yi et al. investigated the extracellular interferon-inducible transmembrane protein 3 (IFITM3) role in the immunological response to *Brucella* infection. For the first time, they demonstrated that *B. melitensis* strain M5 can induce macrophages to release a significant amount of exosomes. The most significant finding was that the exosomes from *Brucella* M5-infected cells effectively reduced *Brucella’s* intracellular survival. Additionally, animals developed *Brucella* antibodies after immunization with exosomes containing IFITM3, reducing spleen CFU and tissue damage. Overall, this provides novel ideas for the progress of candidate vaccines for *Brucella* (224).

### 4.4. Basic techniques for *Brucella* diagnosis

Classical serological tests, such as RBPT and tube agglutination, are simple, rapid, and inexpensive. On the other hand, they produce non-specific reactions with other Gram-negative bacteria, such as *E. coli* O157, *Vibrio cholera*, *Francisella tularensis*, and *Yersinia enterocolitica*, which have antigenic matches to *Brucella*. To avoid these reactions, ELISA tests, mainly iELISA, have been used as a confirmatory test for brucellosis detection. ELISA tests are characterized by the usage of different antigens, enzyme conjugates, and substrates (109). CFT is a very specific test that can detect IgM and IgG1 antibodies. However, antibodies of the IgG2 type impede complement fixation resulting in false negative results (67). A recent comparative serological assay study for brucellosis diagnosis has used 2,317 sera samples [sheep (*n* = 552), goats (*n* = 1,345), and cattle (*n* = 420)]. The results indicated that 189 (8.2%), 191 (8.2%), and 48 (2.1%) tested positive using RBPT, iELISA, and CFT, respectively (225). Nucleic acid tests combine the ability to identify the pathogen with the rapidity of molecular-based assays to provide great performance. In this concern, *Brucella* spp. has been detected using multiplexed PCR and loop-mediated isothermal amplification (LAMP). LAMP is a method of nucleic acid amplification that is significantly rapid with high sensitivity and specificity. In addition, it does not require expensive reagents or instruments, and so it aids in cost reduction (226).

## 5. Future perspectives for controlling bovine brucellosis

### 5.1. Nanotechnology and recent trends for the diagnosis of bovine brucellosis

The development of novel tools for *Brucella* diagnosis is urgently needed for eliminating rampant *Brucella* pathogens in many parts of the world. Meanwhile, the existing methods for brucellosis diagnosis are time-consuming and expensive as they require a weary experimental procedure and sophisticated experimental devices. To overcome these shortcomings, it is truly necessary to establish real-time, on-site, sensitive, and rapid detection methods. For bovine brucellosis, Yang and co-workers investigated a simple, visible, specific, and reliable label-based polymer nanoparticles lateral flow immunoassay (LFIA) biosensor that can reduce the need for advanced equipment and simplify the detection technique. *B. abortus*-LAMP associated with nanoparticle-based LFIA targeting the BruAb2_0168 gene was established and performed successfully, and the technique demonstrated excellent sensitivity and specificity for the detection of many *Brucella* strains, including reference strains, vaccine strains, and field isolates (227). For on-site diagnosis of human brucellosis, LFIA was designed for the first time as a signal probe depending on blue silica nanoparticles (SiNPs) for quick detection with good sensitivity and specificity (228). Li et al. proved that the *Brucella* Multiple Cross Displacement Amplification-Lateral Flow Biosensor (MCDA-LFB) is a fast, easy, reliable, and sensitive method for detecting all *Brucella* spp. strains, and it can be utilized as a possible *Brucella* strain screening tool in many laboratories (229).

Nanotechnology has transformed the field of infectious disease diagnosis and the development of pharmaceutics (230, 231). Their importance in biological applications stems from their reduced size and distinct physicochemical features, which enabled regulated drug release, targeted drug delivery, and *in vivo* immunomodulation. In terms of sensitivity, specificity, time, and cost, nanotechnology has been proved to be a more efficient diagnostic tool. Metal nanoparticles, polymeric nanoparticles, nanoemulsions, liposomes, and nanocrystals are among the nanomaterials employed in veterinary diagnostics and therapies (232). Hybridization assays based on metal nanoparticles are simple to set up and can be read visually via a color shift caused by plasmonic interactions between the probes, so they are interesting approaches for diagnostic purposes. In particular, gold has been preferred in the application of these hybridization assays for the molecular detection of *Brucella* pathogens. Usually, with the addition of simple reagents such as salts, gold causes a color shift from red to purple when aggregated (233). Pal et al. have demonstrated the visual detection of *Brucella* in bovine biological samples using DNA-activated gold nanoparticles. They created a thiol-modified probe that was specific to a certain gene that codes for *Brucella* outer membrane protein. More importantly, they proved that the gold nanoparticle-based probe can be used for direct and simple visual identification of *Brucella* antigen from a wide range of bovine materials, including sperm, milk, and urine. Additionally, this test could be used to choose bulls for semen collection, to examine frozen semen before artificial insemination, or to examine bulk milk before packaging to ensure customer safety by decreasing exposure to *Brucella* pathogens (234).

A rapid and affordable method for diagnosing human brucellosis has been created using a gold nanobiosensor based on the localized surface plasmon resonance (LSPR). LPS of *B. melitensis* and *B. abortus* were isolated and covalently attached to the surface of gold nanoparticles for this purpose. The cutoff value for detecting captured anti-*Brucella* antibodies was determined by measuring the redshift on the LSPR peak, which revealed a significant difference between the healthy and true positive patients. Moreover, for an accurate assessment of this method, 40 sera from true negative and positive patients were used for comparing its results with the culture (standard method), standard tube agglutination test, and anti-brucellosis IgM and IgG levels using ELISA. The LSPR-based technique was accurate with 85, 100, 100, and 86% in terms of sensitivity, specificity, positive predictive value, and negative predictive value, respectively (235). In cattle, a comparative study was performed to evaluate the use of gold nanoparticles (GNPs) and quantum dots (QDs) as labels in the immunochromatographic serodiagnosis of brucellosis. For QDs, the results were highly sensitive in detecting specific antibodies against *B. abortus* LPS. So, the use of QDs in immunochromatographic serodiagnosis proved to be a good technique to increase the accuracy of immunochromatographic assay (ICA) (236).

The sensitivity and specificity for detecting *Brucella* pathogens have recently been increased using biosensors. They can turn biological responses into electrical signals for analysis. A bioreceptor (antibodies, enzymes, nucleic acids, microbes, etc.) is combined with a physical transducer (optical, electrochemical, or mass-based) to generate a measurable signal (237). Many new signal transduction systems based on nanoparticles have been developed to improve pathogen detection strategies (237, 238). Sandwich immunoassay nanoparticle-based biosensors are regarded as a promising way to introduce selective and sensitive diagnostic tools (239). Silica and magnetic nanoparticles have distinct properties linked to their hydrophobic surfaces and the capacity to modify their surfaces with chemical groups, and so they were used for visual and spectrophotometric detection of *Brucella* (240–242). An immunosensor was designed based on blue-SiNPs and paramagnetic nanoparticles (PMNPs). The synthesized immunosensor was conjugated with a polyclonal antibody against *B. abortus* and applied to the bacterial culture. Then, a magnet was used to create a sandwich structure of PMNPs *B. abortus*-blue-SiNPs. Afterward, the absorbance of the blue color produced by the silica structure was quantified using a spectrophotometer, combined with the visible color change, to determine the presence of bacterial cells in the samples (243). Li et al. developed another immunosensor using the immune magnetic beads (IMBs) probe and the QDs staphylococcal protein A (SPA) probe. The IMB probe and serum were combined first to allow the *Brucella* antibodies to interact with *Brucella* outer membrane protein coated on the IMB probe surface. The fluorescence intensity of QDs was correlated with the quantity of anti-*Brucella* antibodies. For rapid and label-free detection of *B. melitensis* in milk and milk products, a new quartz crystal microbalance (QCM) aptasensor has been developed. On the QCM chip, the particular aptamer sequence linked with magnetic nanoparticles was immobilized to identify *B. melitensis* (244). A rapid, accurate, simple, and sensitive fluorescent immunochromatographic strip test (ICST) based on a quantum dot fluorescent microsphere (QDFM) is another application for the field screening of brucellosis using animal serum.

The impedance technique is another rapid and inexpensive alternative to label-free biosensor technologies. For quick detection of *B. melitensis*, Wu et al. developed a label-free impedance immunosensor based on gold nanoparticles with modified screen-printed carbon electrodes (GNP-SPCEs). The interaction of *B. melitensis* antigens on the surface of GNP-SPCEs with antibody molecules was studied using cyclic voltammetry (CV) and electrochemical impedance spectroscopy (EIS). In less than 1.5 h, this biosensor was able to detect 1 × 10^4^ and 4 × 10^5^ CFU/ml of *B. melitensis* in pure culture and milk samples, respectively (245). Zinc oxide nanoparticles (ZnO-NPs) are novel types of low-cost, low-toxicity nanomaterials. In this concern, a *Brucella* identification biosensor has been designed for detecting *Brucella* in milk samples (246).

### 5.2. Nanotechnology for treatment of bovine brucellosis

Many host immunological responses, such as lysosomal enzymes and oxidative burst (247), cannot interact with the intracellular *Brucella*, and medications are unable to effectively target *Brucella*-infected cells as they lose their antimicrobial effectiveness in the intracellular environment (248). In humans, routine treatment regimens for brucellosis by using two or more antibiotics are the most effective way to evade relapses occurring and prevent prolonged use of these drugs (249). Khan et al. revealed that mutations in the rpoB, gyrA, and gyrB genes have been linked to antimicrobial resistance in rifampicin and ciprofloxacin (250). Resistance to rifampin, ampicillin–sulbactam, trimethoprim–sulfamethoxazole, and ampicillin in *B. melitensis* was estimated at 36.36, 31.82, 27.27, and 22.70%, respectively. Resistance to rifampin, trimethoprim–sulfamethoxazole, ampicillin, and ampicillin–sulbactam was detected at 35.71, 32.14, 28.57, and 32.14% of *B. abortus* isolates, respectively (212).

Gene therapy, which is beneficial in the clinic, may provide an alternate path to a complete cure for brucellosis by assisting in the deletion or inactivation of genes involved in *Brucella* multiplication in host cells. To simulate the host microorganism interaction *in vitro*, ovine macrophages were infected with *B. melitensis* and then the infected cells were transduced with CRISPR/Cas9 lentiviral vectors targeting *Brucella* RNA polymerase subunit A (RpolA) or virulence-associated gene (virB10) at a multiplicity of infection (MOI) of 60. When infected cells were transduced with the RpolA vector, the number of internalized *Brucella* per cell was decreased significantly, whereas when macrophages were transduced with a conventional lentiviral vector expressing the green fluorescence protein, the number of internalized brucellae per cell remained unaffected. This highlights the bactericidal effect of the CRISPR/Cas9 system (251).

Niosomes are drug-delivery materials made from cholesterol and a non-ionic surfactant that self-assemble (252, 253). They improve medication bioavailability by delaying drug release and changing drug half-lives, resulting in good drug accumulation within the targeted site (254). Chitosan and genetically modified organisms (GMOs) are minimal in toxicity and highly compatible with biological systems (255). The synergistic effect of chitosan/GMO is beneficial for drug targeting and maintenance by increasing the mucoadhesion characteristics of niosomes (254, 256). Abo EL-Ela et al. showed that the combination of doxycycline (DOX)-loaded chitosan–sodium alginate nanoparticles (257, 258) with *in situ* pH-responsive curcumin-loaded niosome hydrogel for brucellosis treatment and splenic count reduction is an effective recipe. The successful production of DOX and curcumin as nanomaterials for the treatment of intracellular bacteria may improve human brucellosis therapy due to their prolonged release, stability, and high trapping efficiency. However, the injected antimicrobial drugs did not completely eliminate *Brucella* in the artificially infected Guinea pigs. The results showed a considerable drop in viable *Brucella* numbers in the spleen and blood (259).

Using RB51 phage lysates (RL) and S19 lysates (SL), researchers have described unique and successful immunotherapy for the treatment of bovine brucellosis in cows. The combination of these two phage lysates (RL and SL) was administered subcutaneously in a 2-mL dose, and blood samples were found to be *Brucella*-free even after 3 months of phage cocktail immunization. RL elicited a stronger cell-mediated immune response than SL, while SL elicited a higher amount of humoral immunological response. The study’s findings are encouraging, suggesting that bacteriophage lysates could be used to treat bovine brucellosis because the conventional treatment regimens are ineffective (260).

Another study has produced polyanhydride nanoparticles of copolymers of sebacic acid to encapsulate the antibiotics doxycycline and rifampicin, and the best antibacterial activity was reported at 72 h and lasted up to a week after the nanoparticles released rifampicin for a week. In comparison with soluble drug controls, treatment of *B. melitensis*-infected macrophages with rifampicin-containing nanoparticles rapidly removed viable intracellular bacteria after 48 h, and pretreatment with the nano-formulations prevented intracellular infection (261–263). Infected BALB/c mice were treated for 5 days with a nanoparticle cocktail combining doxycycline and rifampicin, which reduced bacterial burden in the liver. Infected mice with *B. melitensis* were given either a daily 0.5 mg doxycycline dose or a single 0.5 mg doxycycline-encapsulated nanoparticles delivered once a week to demonstrate the *in vivo* extended antibiotic release. After 3 weeks, bacterial numbers in the spleen and liver of animals treated with the weekly nano-formulation and other animals that received daily soluble medication were statistically equivalent, indicating a 7-fold dosage sparing. These findings showed that using nanotherapeutics to treat persistent bacterial infections increased antimicrobial efficacy and improved *in vivo* activity through a combination of intracellular transport, dosage sparing, and prolonged release (264).

### 5.3. Methods for eradication of bovine brucellosis

The major goal during the settlement of *Brucella* control programs is decreasing the spread of microorganisms. Hosein et al. carried out a study measuring the time needed to reach *Brucella*-free herd status. They found that 6 months are required to reach free status, which is considered a long time, allowing infection to spread to other areas, especially under unsanitary conditions, a husbandry system that favors mixed populations of different ages, sex, aborted, and pregnant animals, and a lack of controlled animal movement. As a result, efficient animal brucellosis control needs surveillance to identify diseased animal herds, reservoir eradication, and vaccination of young heifers (64). In Dohuk Governorate, Iraq, the usage of the Rev. 1 vaccine for all female and male animals older than 3 months reduces the prevalence of brucellosis from 36 to 6.6% (23). The results revealed that successful animal vaccination, gradual culling of seropositive cattle, and small ruminants through slaughter, environmental hygiene, and human personal protection all have a role in limiting disease spread in livestock and human populations (265, 266), in addition to the eradication of carrier animals in the herd, such as dogs, cats, and mice, to eliminate infection sources (267).

Veterinary organizations should also use ongoing education and training initiatives to raise farmers’ knowledge and understanding of prevention methods and transmission routes as an important principle (91, 268). It is also worth noting that brucellosis risks are higher in dairy farms that use artificial insemination or natural breeding with non-certified brucellosis bulls, so it is important to use semen from certified free farms (97).

In the point of test and slaughter in most developed countries, culling of the suspected or reactor animals based on serology is practiced in addition to immunization. In most developing countries, this technique is not implemented due to financial constraints and the lack of healthcare infrastructure. A production strategy that avoids direct or indirect contact with diseased neighboring farms and/or contaminated feed or pasture is also essential as a part of the control plan (33). When the rate of infection is reduced to an acceptable level, a test and slaughter strategy could be implemented. So, if the disease prevalence is quite low (1 to 4%), then eradication of the disease in a short-to-medium timeframe could be achieved using a test and slaughter eradication program. At the second level, if the prevalence is low to moderate (5–10%) then eradication of the disease in a medium-long timescale could be achieved by simultaneous vaccination of young replacements as well as testing and slaughter of seropositive adult animals. At the third level, if there is a high rate of occurrence (10%), then the mass vaccination program is the only way to control the disease, regardless of the level of professional organization or financial resources available (29). Moreover, Pasteurization renders *B. abortus* inactive, and its survival outside the host is primarily dependent on environmental factors. *Brucella* can live for 2–3 months in wet soil, up to 8 months in an aborted fetus in the shade, 1–2 months in dry soil, 3–4 months in fecal matter, and up to 8 months in liquid sewage tanks. Bacterial viability is prolonged at cold temperatures, and organisms can survive in the frozen cadaver for many years (269). Raw or undercooked animal items (even bone marrow) and unpasteurized dairy products should not be ingested (270).

## 6. Conclusion

Brucellosis is regarded as one of the most hazardous pathogens and seriously affects humans and terrestrial animals. It is still a serious threat to animals and humans despite all the measures taken by the WHO and veterinary authorities with a significant economic effect all over the world. In the current study, we provided an updated insight into the worldwide *Brucella* distribution, possible predisposing factors incorporated in emerging *Brucella* pathogens, immune status and different types of *Brucella* vaccines (subunit vaccines, vector vaccines, and genetic engineering vaccines), genomics and proteomics approaches, and future perspectives for prevention and control of bovine brucellosis, which undoubtedly will be used as a platform for controlling *Brucella* pathogens in many endemic countries; this will help scientists, veterinarians, and animals’ owners to prevent introducing *Brucella* pathogens to the free areas. More specifically, many risk and predisposing factors have been summarized, including the control of fetal fluids, placenta, dogs and cats, and animal movements as the introduction of new animals to herds without certification. Moreover, pasteurization of dairy products should be done and watched as it is an important source of transmitting the disease to humans. The veterinary authorities should educate farmers to be aware of the dangers of the disease, how it can be transmitted between animals and humans, and how they can protect themselves. An important section of this review has discussed the novel techniques based on nanotechnology and genetic engineering of the antigenic structures of the pathogen to make an easy, simple, and proper diagnosis. Finally, we conclude that following many comprehensive studies in the field of genomics and proteomics of *Brucella* pathogens, vaccination proved to be effective for defeating these pathogens in the endemic areas as well as preventing disease introduction to the other free areas of the world.

## Author contributions

ASD: Conceptualization, Data curation, Writing – original draft, Writing – review & editing. AlE: Conceptualization, Data curation, Investigation, Writing – original draft. MN: Investigation, Supervision, Review & editing. AS: Investigation, Visualization & editing. AG: Investigation, Supervision, Review & editing. GZ: Conceptualization, Writing – review & editing. SAA: Conceptualization, Investigation, Writing – review & editing. AZ: Visualization, Writing – review & editing. MZ: Investigation, Review & editing. AhE: Conceptualization, Review & editing. WM: Visualization, Review & editing. WL: Funding acquisition, Supervision, Writing – review & editing.
