# Supplementary material for: Brucellae as resilient intracellular pathogens: epidemiology, host–pathogen interaction, recent genomics and proteomics approaches, and future perspectives
Source: Front Vet Sci. 2023 Oct 9;10:1255239. doi: 10.3389/fvets.2023.1255239 (PMC10591102; doi:10.3389/fvets.2023.1255239)
Supplement: Supplementary file 1 [file Table_1.pdf]

Table (S1): Worldwide prevalence of brucellosis in humans and different animal species

Brucella species	Prevalence	Host	Area of study	Date of study	Reference
B. abortus	Herd (6.5%). Individual animal (0.05%).	Cattle	Urban and peri-urban areas, Kampala economic zone, Uganda	2011	[1]
Ovine brucellosis	A village flock (0 to 46.8 %).	Sheep	Kafrelsheikh district, Egypt	February to July 2014	[2]
B. melitensis 16M and *B. abortus* S-99	Eight villages (61.5%)	Goats	Central Punjab, Pakistan	July 2017 to July 2018	[3]
	Six villages (75%)	Sheep			
	Seven villages (87.5%)	Buffaloes			
	Eight villages (89%)	Cows			
Human and Bovine brucellosis	10.1%	Humans	North Tongu district, Ghana	February to June 2011	[4]
	22.9%	Cattle			
Brucella spp.	Togo (62.0%)<br>Mali (32.5%)<br>Burundi (14.7%)<br>Cameroon (12.6%)<br>Burkina Faso (3.0%)<br>Senegal (1.3%)<br>Niger (1.2%)	Dairy cattle	West and Central Africa	February 2017 to January 2018.	[5]
Bovine brucellosis	Farm level (10 to 13%)<br>Animal level (4 to 5%)	Cattle	Argentina	2002	[6]
East African community countries					

B. abortus and B. melitensis	0.2% to 21.9%	Cattle	Kenya	2012 to 2014	[7, 8]
	0.0% to 20.0%	Goats			
	0.0% to 13.8%	Sheep			
	0.6% to 35.8%	Humans			
	0% to 11.1%	Camels			
Human brucellosis	6.1% and 25%	Humans	Rwanda	2017	[9]
Bovine brucellosis	29.3% to 31.9%	Cattle	South Sudan	December 2015 to May 2016	[10]
	23.3% to 33.3%	Humans			
B. abortus	0.2% and 11.7%	Cattle	Tanzania	August 2015 to January 2016	[11]
	0% and 2.0%	Goats		September 2012 to July 2013	[12]
	Buffaloes: 7.9%	Buffaloes			
B. abortus and *B. melitensis*	1.2% to 43.8%	Cattle	Uganda	June to September 2014	[13]
	0.3% to 9.8%	Goats			
	4.0% to 33.0%	Humans			
	2.6% to 10.5%	Sheep		October 2009 to March 2010	[14]
	0.0% to 0.2%	Pigs		December 2012 to July 2013	[15]
Southeast Asia					
B. abortus	1% to 2%	Cattle	Thailand and Indonesia	2014	[16]
	4% to 5%		Malaysia and Myanmar		
B.melitensis	1%		Malaysia and Thailand		
B. abortus and *B. melitensis*	5.55% to 78.8% in females.<br>2.23% in males.	Goats	Anhui province is situated on the east side of China.	March to June 2018	[17]
B. melitensis	0 to 11.85%	Humans	Inner Mongolia, China	2012 to 2016	[18]

Brucella spp.	Africa (1.7%)<br>America (16.5%)<br>Asia (0.5%)<br>Europe (17.4%)<br>Ocean (6.0%)	Pigs	Worldwide	January 2000 to January 2020	[19]
Human brucellosis	(15.6%)	Humans	Namibe province, Angola	2012	[20]

1. Makita K, Fèvre EM, Waiswa C, Eisler MC, Thrusfield M, Welburn SC (2011) Herd prevalence of bovine brucellosis and analysis of risk factors in cattle in urban and peri-urban areas of the Kampala economic zone, Uganda. BMC Vet Res. https://doi.org/10.1186/1746-6148-7-60

2. Hegazy Y, Elmonir W, Abdel-Hamid NH, Elbauomy EM (2016) Seroprevalence and "Knowledge, Attitudes and Practices" (KAPs) survey of endemic ovine brucellosis in Egypt. Acta Vet Scand 58:1–7

3. Saeed U, Ali S, Latif T, et al (2020) Prevalence and Spatial Distribution of Animal Brucellosis in Central Punjab, Pakistan. Int J Environ Res Public Health 17:1–14

4. Tasiame W, Emikpe BO, Folitse RD, Fofie CO, Burimuah V, Johnson S, et al.. The prevalence of brucellosis in cattle and their handlers in north tongu school of veterinary medicine, college of health sciences, kwame nkrumah university of science. Afr J Infect Dis. (2016) 10:111–7. 10.21010/ajid.v10i2.6

5. Musallam I, Ndour AP, Yempabou D, et al (2019) Brucellosis in dairy herds: A public health concern in the milk supply chains of West and Central Africa. Acta Trop. https://doi.org/10.1016/J.ACTATROPICA.2019.105042

6. Samartino LE (2002) Brucellosis in Argentina. Vet Microbiol 90:71–80

7. Kairu-Wanyoike S, Nyamwaya D, Wainaina M, et al (2019) Positive association between Brucella spp. seroprevalences in livestock and humans from a cross-sectional study in Garissa and Tana River Counties, Kenya. PLoS Negl Trop Dis 13:e0007506

8. Chota AC, Magwisha HB, Stella B, Bunuma EK, Shirima GM, Mugambi JM, Omwenga SG, Wesonga HO, Mbatha P, Gathogo S (2016) Prevalence of brucellosis in livestock and incidences in humans in east Africa. African Crop Sci J 24:45–52

9. Njunwa KJ, Ruhirwa R, Kiiza G, et al (2017) Seroprevalence of Brucellosis among Patients Attending a District Hospital in Rwanda. Am J Trop Med Hyg 97:831–835

10. Madut NA, Ocan M, Muwonge A, Muma JB, Nasinyama GW, Godfroid J, Jubara AS, Kankya C (2019) Sero-prevalence of brucellosis among slaughterhouse workers in Bahr el Ghazal region, South Sudan. BMC Infect Dis 19:1–7

11. Sagamiko FD, Muma JB, Karimuribo ED, Mwanza AM, Sindato C, Hang'ombe BM (2018) Sero-prevalence of Bovine Brucellosis and associated risk factors in mbeya region, Southern highlands of Tanzania. Acta Trop 178:169–175

12. Assenga JA, Matemba LE, Muller SK, Malakalinga JJ, Kazwala RR (2015) Epidemiology of Brucella infection in the human, livestock and wildlife interface in the Katavi-Rukwa ecosystem, Tanzania. BMC Vet Res 11:1–11

13. Nguna J, Dione M, Apamaku M, et al (2019) Seroprevalence of brucellosis and risk factors associated with its seropositivity in cattle, goats and humans in Iganga District, Uganda. Pan Afr Med J. https://doi.org/10.11604/PAMJ.2019.33.99.16960

14. Lolli C, Marenzoni ML, Strona P, Lappo PG, Etiang P, Diverio S (2016) Infections and risk factors for livestock with species of Anaplasma, Babesia and Brucella under semi-nomadic rearing in Karamoja Region, Uganda. Trop Anim Health Prod 48:603–611

15. Erume J, Roesel K, Dione MM, et al (2016) Serological and molecular investigation for brucellosis in swine in selected districts of Uganda. Trop Anim Health Prod 48:1147–1155

16. Zamri-Saad M, Kamarudin MI (2016) Control of animal brucellosis: The Malaysian experience. Asian Pac J Trop Med 9:1136–1140

17. Rahman SU, Zhu L, Cao L, Zhang Y, Chu X, Feng S, Li Y, Wu J, Wang X (2019) Prevalence of Caprine brucellosis in Anhui province, China. Vet World 12:558–564

18. Liu Z guo, Wang M, Ta N, Fang M gang, Mi J chuan, Yu R ping, Luo Y, Cao X, Li Z jun (2020) Seroprevalence of human brucellosis and molecular characteristics of Brucella strains in Inner Mongolia Autonomous region of China, from 2012 to 2016. Emerg Microbes Infect 9:263–274

19. Gong QL, Sun YH, Yang Y, et al (2021) Global Comprehensive Literature Review and Meta-Analysis of Brucella spp. in Swine Based on Publications From 2000 to 2020. Front Vet Sci 8:630960

20. Mufinda FC, Boinas F, Nunes C (2017) Prevalence and factors associated with human brucellosis in livestock professionals. Rev Saude Publica. https://doi.org/10.1590/S1518-8787.2017051006051
